# PRMT5‐Dependent Stabilization of VPS34 Orchestrates Copper Trafficking to Shield Cancer Cells from Cuproptosis and Radiotherapy

**DOI:** 10.1002/advs.76350

**Published:** 2026-07-08

**Authors:** Wei Chen, Ming‐Ying Xiao, Lan‐Lan Guo, Hao‐Yue Hu, Sui‐Xian Zhang, Bai‐Qiang Dong, Chen‐Fei Wu, Xia‐Hang Huang, Li Li, Run‐Zhe Chen, Yuan‐Yang Huang, Ying Jin, Hai Song, Ying Wang, Qi‐Wen Li, Ming Chen, Xuan Li, Yuan‐Yuan Chen

**Affiliations:** ^1^ Department of Radiation Oncology Sun Yat‐sen University Cancer Center Guangzhou P. R. China; ^2^ State Key Laboratory of Oncology in South China, Collaborative Innovation Center For Cancer Medicine Sun Yat‐sen University Cancer Center Guangzhou P. R. China; ^3^ United Laboratory of Frontier Radiotherapy Technology Sun Yat‐Sen University Chinese Academy of Sciences Ion Medical Technology Co. Guangzhou Guangdong P. R. China; ^4^ Zhejiang Cancer Hospital, Hangzhou Institute of Medicine (HIM) Chinese Academy of Sciences Hangzhou Zhejiang China; ^5^ Department of Respiratory and Critical Care Medicine, the Fourth Affiliated Hospital of School of Medicine, and International School of Medicine International Institutes of Medicine, Zhejiang University Yiwu China

**Keywords:** copper homeostasis, cuproptosis, PRMT5, radiotherapy, resistance, VPS34

## Abstract

Targeting cuproptosis for cancer therapy is hindered by an incomplete understanding of the intrinsic defense networks through which tumor cells mobilize to evade this fate, particularly under therapeutic stress such as radiotherapy. Here, we identify VPS34‐mediated copper homeostasis as a critical anticuproptotic process through a genome‐wide CRISPR activation screen. PRMT5 directly methylated VPS34 at Arg174 and inhibited K48‐linked polyubiquitination and proteasomal degradation of VPS34 by recruiting the deubiquitinase USP10. This stabilization increased the cuproptotic defense of tumor cells by promoting autophagic degradation of the copper importer SLC31A1 and plasma membrane translocation of the exporter ATP7A, thereby reducing the intracellular copper load. Functional analysis and clinical correlation revealed that the PRMT5‐VPS34 axis is robustly activated by radiation stress and is necessary for adaptive radioresistance. The inhibition of PRMT5 or VPS34 expression suppressed tumor growth and enhanced the sensitivity of tumors to radiotherapy by unleashing latent copper cytotoxicity both in vitro and in vivo. Together, our findings identify the PRMT5‐VPS34 axis as a therapeutic target for sensitizing refractory cancers to radiotherapy.

## Graphical abstract

Radioresistance arises partly from tumor cells evading cuproptosis via unknown defenses. This study reveals that the PRMT5‐VPS34 axis acts as a radiation‐activated anticuproptotic mechanism: PRMT5 methylates VPS34 at Arg174, recruiting USP10 to remove K48‐linked ubiquitination and prevent degradation. Stabilized VPS34 lowers copper levels by degrading importer SLC31A1 and relocating exporter ATP7A, conferring adaptive radioresistance. Targeting this axis unleashes copper cytotoxicity and sensitizes refractory tumors to radiotherapy.

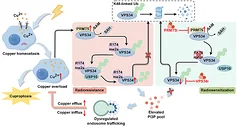

## 1 Introduction

Copper is an essential micronutrient that serves as a catalytic cofactor for enzymes that govern fundamental biological processes [1]. However, intracellular copper levels must be strictly circumscribed, as free copper overload triggers cytotoxicity through oxidative stress and proteotoxic aggregation. Recently, a distinct form of regulated cell death termed “cuproptosis” has been characterized, driven by the direct binding of copper to lipoylated components of the tricarboxylic acid (TCA) cycle [2, 3]. While the induction of cuproptosis holds promise as a therapeutic strategy for cancers, including as a potential means to overcome therapeutic resistance, cancer cells frequently exhibit remarkable adaptive plasticity [4, 5], allowing them to adapt to imbalances in metal ions. Despite the growing understanding of cuproptosis triggers [2, 6], the intrinsic homeostatic machinery through which tumor cells mobilize to sense copper pressure and evade this specific form of cell death remains largely elusive. Moreover, whether dysregulated copper homeostasis contributes to the adaptive resistance of tumors to conventional treatments such as radiotherapy is poorly understood.

Cellular copper homeostasis is primarily maintained by a delicate equilibrium between uptake, mediated by the high‐affinity transporter SLC31A1 (CTR1), and efflux, facilitated by Cu‐transporting ATPases such as ATP7A [7]. The turnover and membrane localization of these transporters are tightly regulated by the endolysosomal trafficking system [8]. To systematically identify key host factors that confer resistance to copper toxicity and to elucidate potential links between copper homeostasis and therapeutic adaptation, we performed a genome‐wide CRISPR activation (CRISPRa) screen. This unbiased approach identified VPS34 (PIK3C3), the sole class III phosphatidylinositol 3‐kinase (PI3K), as a key candidate essential for cell survival under copper stress. As a central regulator of autophagy and endosomal trafficking [9, 10], VPS34 controls the sorting of membrane proteins. However, whether and how VPS34 orchestrates the trafficking of copper transporters to prevent cuproptosis have not been established.

Protein arginine methyltransferase 5 (PRMT5) is a major type II methyltransferase that governs genome stability and cell survival by modulating the posttranslational modification (PTM) of diverse substrates [11]. High PRMT5 expression is frequently observed in aggressive malignancies and is correlated with poor prognosis and resistance to radiotherapy, suggesting that PRMT5 plays a central role in therapeutic adaptation [12, 13]. In this study, we identified PRMT5 as a critical upstream regulator of VPS34 that governs cellular sensitivity to copper stress. We demonstrate that PRMT5 methylates VPS34 at Arg174 (R174me2s), which facilitates the recruitment of the deubiquitinase USP10. This interaction prevents the K48‐linked polyubiquitination and proteasomal degradation of VPS34, thereby maintaining the intracellular abundance of VPS34 required for efficient copper efflux. Consequently, this PRMT5‐VPS34 signaling axis confers adaptive resistance to copper‐induced cell death and, importantly, to radiotherapy. These findings suggest that targeting this pathway represents a promising strategy to sensitize tumors to treatment via copper toxicity.

## 2 Experimental Section

### 2.1 Cell Lines and Cell Culture

Human embryonic kidney cells (HEK293T) were purchased from American Type Culture Collection (ATCC). Small lung cancer cells (SBC‐2 and SW1271) were generously provided by Prof. Ming Chen at ZheJiang Cancer Hospital. HEK293T and SW1271 cells were grown in DMEM (C11995500BT; Gibco, Grand Island, NY, USA) supplemented with 10% FBS (10099141; Gibco, Grand Island, NY, USA), and SBC‐2 cells were grown in RPMI 1640 medium (C11875500BT; Gibco, Grand Island, NY, USA) supplemented with 10% FBS (10099141; Gibco, Grand Island, NY, USA). All the cell lines were maintained at 37°C and 5% CO2 and were regularly tested for mycoplasma.

### 2.2 Reagents and Antibodies

Elesclomol (S1052), tetrathiomolybdate (E1166), rotenone (S2348), oligomycin A (S1478), FCCP (S8276), bafilomycin A1 (S1413), cycloheximide (S7418), chloroquine (S6999), pemrametostat (S8664) and VPS34 inhibitor 1 (S8456) were purchased from Selleckchem (Houston, TX, USA). Puromycin (02190146) was purchased from MP Biomedicals (Irvine, CA, USA). An anti‐SQSTM1/p62 (sc‐28359) antibody was purchased from Santa Cruz Biotechnology (Dallas, TX, USA), and an anti‐LC3 (NB100‐2220) antibody was purchased from Novus Biologicals (Centennial, CO, USA). Antibodies against EEA1 (28347‐1‐AP), RAB7A (55469‐1‐AP), LAMP1 (21997‐1‐AP), MYC‐Tag (16286‐1‐AP), Flag‐Tag (20543‐1‐AP), GAPDH (10494‐1‐AP) and alpha tubulin (66031‐1‐Ig), as well as horseradish peroxidase (HRP)‐conjugated goat anti‐mouse IgG (SA00001‐1) and HRP‐conjugated goat anti‐rabbit IgG (SA00001‐2), were purchased from Proteintech (Rosemont, IL, USA). Antibodies against VPS34 (S4623), SDMA (13222), HA‐Tag (3724S), K48‐linked polyubiquitin (8081S) and K63‐linked polyubiquitin (5621S) were purchased from Cell Signaling Technology (Danvers, MA, USA). Antibodies against ATP7A (ab131400) and lipoic acid (ab58724) were purchased from Abcam (Cambridge, MA, USA). An Alexa Fluor 488‐conjugated secondary antibody (A‐11008) and Phalloidin‐ and Alexa Fluor 647‐conjugated secondary antibodies (A‐31571) were purchased from Thermo Fisher Scientific (Waltham, MA, USA).

### 2.3 Plasmids

Human PRMT5 and VPS34 (full‐length and R174K mutant) were cloned and inserted into the pSin‐EF2 vector. The pRK5‐HA‐Ubiquitin‐WT (17,608), pRK5‐HA‐Ubiquitin‐K48 (17,605), and pRK5‐HA‐Ubiquitin‐K63 (17606) plasmids were obtained from Addgene. The pCMV‐USP10 (human)‐3×Myc‐Neo (P53606) plasmid was acquired from MiaoLingBio (China). Short hairpin RNAs targeting PRMT5 (5’‐AGGGACTGGAATACGCTAATT‐3’) were cloned and inserted into the pLVX‐TRE3G‐IRES vector (113405; Addgene). All the newly constructed plasmids were verified via DNA sequencing. Transfection of the plasmids was performed using Lipofectamine 3000 (Thermo Fisher Scientific) according to the manufacturer's instructions.

### 2.4 SiRNA

The targeted siRNA sequences used in this research were as follows:

siNC: 5’‐ UUCUCCGAACGUGUCACGUTT ‐3’;
siPRMT5‐1: 5’‐GGUGAACACAGUACUACAUTT‐3’;
siPRMT5‐2: 5’‐CGAAAUAGCUGACACACUA‐3’;
siVPS34‐1: 5’‐CCCAUGAGAUGUACUUGAACGUAAU‐3’;
siVPS34‐2: 5’‐ ATGGCTGAAACTACCAGTAAA ‐3’;
siUSP7: 5’‐ CCTGGATTTGTGGTTACGTTA ‐3’;
siUSP10: 5’‐ CCCUGAUGGUAUCACUAAAGA ‐3’;
siUSP16: 5'‐ CCGGAAAUCUUAGAUUUGGCUCCUU ‐3';
siUSP22: 5’‐ AGCTACCAGGAGTCCACAAAG ‐3’;
siUCHL5: 5’‐ TGAAGGTGAAATTCGATTTAA ‐3’;
siSENP3: 5`‐ AGAAAUCGCUGGUAUAAAUUU ‐3`; and
siUSP9X: 5’‐CAAUAAGGAGCUACUGCUAdTdT‐3’;
siFDX1: 5’‐GCAATCACTGATGAGGAGAAT‐3’.

All siRNAs were synthesized by GenePharma and transfected into cells using siRNA‐mate (G04003‐1) according to the manufacturer's instructions.

### 2.5 Genome‐Wide CRISPR Activation Screen and Data Analysis

SBC‐2 cells expressing dCas9‐VP64 were generated by transducing the lentiviral vector lenti_dCAS9‐VP64_blast (Addgene #61425). Prior to screening‐scale transduction, dCas9‐VP64 cell lines were selected with blasticidin. SBC‐2 cells expressing dCas9‐VP64 (2.5 × 108) were transduced with a pooled genome‐wide lentiviral sgRNA library (Addgene #92379, #92380) at a multiplicity of infection of 0.3. Stably transduced cells were selected with 2 µg per ml puromycin, and 4 × 107 cells were passaged every 24–48 h at a density of 2 × 106 cells per 10‐cm dish in growth medium for the duration of the screen. At 7 d after puromycin selection, 1.5 × 108 cells were treated with increasing doses of elesclomol (40 nm) for 72 h. Afterward, 1 × 107 cells were collected from the surviving population of elesclomol‐treated cells and an endpoint‐matched untreated population.

Genomic DNA was extracted from the collected cells, and next‐generation sequencing libraries were constructed using a two‐step PCR method. For the genomic DNA samples, the reaction volume was determined to ensure coverage of 2,000 sgRNAs per reaction. Universal primers containing bridge sequences targeting the sgRNA region were designed, and the first PCR step was performed using high‐fidelity NEB Q5 enzyme to amplify the target fragments. PCR products were pooled and purified using magnetic beads. A second PCR was conducted using 15 µL of the purified product with index primers to attach specific barcodes. The final library products were purified via magnetic beads, validated by agarose gel electrophoresis (band size ∼280 bp), and quantified using a Qubit instrument to a concentration of 3 ng/µL.

The libraries were sequenced on an Illumina HiSeq Xten/Novaseq platform using the PE150 paired‐end mode. For data analysis, paired‐end reads were merged using FLASH software. Target sgRNA sequences were extracted from the merged reads using an in‐house script based on 5 bp upstream and downstream flanking sequences, followed by data volume statistics and quality assessment. The extracted sgRNAs were aligned to the reference library using MAGeCK software. Read counts were normalized using the control‐sgRNA method. To identify candidate genes associated with elesclomol resistance, differential analysis between the treated and untreated groups was performed using the MAGeCK robust rank aggregation (RRA) algorithm. Functional enrichment analyses were subsequently performed on the significant hits using Metascape [14].

### 2.6 CRISPR‐Cas9‐Mediated Gene Knock Out

The CRISPR‐Cas9 system was used to knock out PRMT5 and VPS34. Single guide RNA was cloned and inserted into the empty backbone of lenti‐CRISPR v2.

The following sgRNA sequences were used in this research:

sgPRMT5‐1: 5’‐GACTGGAATACGCTAATTGT‐3’;
sgPRMT5‐2: 5’‐CAGGAGAGTGAGTCTCTGGA ‐3’;
sgVPS34‐1: 5’‐TATGGGATGTGTATGGTCCC ‐3’; and
sgVPS34‐2: 5’‐GTTTATCCAGTCAGTTCCTG ‐3’.

The sgAAVS sequence (5’‐CACCGTCCCCTCCACCCCACAGTG‐3’) was used as a negative control.

Plasmids containing the sgRNA sequences were transfected into HEK293T cells with the psPAX2 packaging plasmid and pMD2. G envelope‐expressing plasmid. SBC‐2 cells were virally infected to express sgRNA and selected with puromycin for 3 days to generate stable cell lines.

### 2.7 Copper Measurement

Cells were seeded on 6‐well plates and subsequently treated with the indicated agents. After the agents were removed, the treated cells were incubated with 5 µm Coppersensor‐1 for 15 min in the dark at 37°C. Afterward, the cells were washed with PBS and analyzed using CytExpert software on a cytoFLEX S (Beckman Coulter). A minimum of 5,000 single cells were analyzed per well.

For the ICP‒MS analysis, a series of cell pellets were prepared and subjected to acid digestion. Each sample was weighed and placed into a calibrated digestion tube. To each tube, 10 mL of nitric acid and 0.5 mL of high‐chloric acid were added. The samples were then subjected to a controlled digestion process on an adjustable electric hot plate under the following conditions: initial heating at 120°C for 0.5 to 1 h, then a gradual increase to 180°C, which was maintained for 2 to 4 h, and a final increase to 200°C to 220°C. The digestion was continued until the solution turned colorless or slightly yellow, indicating reaction completion. The digested samples were then cooled and brought to a fixed volume of 20 mL with deionized water and mixed thoroughly for subsequent analysis. A standard series for copper was prepared by accurately pipetting varying volumes of a 1 mg/L copper standard solution into 100 mL volumetric flasks. Volumes of 0, 0.2, 0.5, 1.0, 1.5, and 2.0 mL were used, resulting in copper concentrations of 0.0, 2.0, 5.0, 10.0, 15.0, and 20.0 µg/L, respectively. Each standard solution was brought to volume with nitric acid and mixed well to ensure homogeneity. The copper content in the samples was determined using a graphite furnace atomic absorption spectrometer (Model Shanghai Spectro SP‐3802AA) a wavelength of 324.8 nm. The instrument was calibrated using the prepared standard solutions, and the absorbance readings were recorded within a specified concentration range where the relationship between the absorbance and the copper concentration was linear. The concentration of copper in the samples was calculated using the calibration curve method. The formula used for the calculation is as follows:

$$X\left(\mathrm{ng}/10^{6}\right)=\frac{\left(C-C_{0}\right)\times V\times f}{m}$$

where X is the concentration of copper in the sample, C is the concentration of copper in the sample mixture, C_0_ is the concentration of the sample mixture, V is the final volume of the sample mixture after dilution, f is the dilution factor, and m is the number of cells in the sample, expressed in millions (10^6^).

### 2.8 Immunoblot Analyses

For the analysis of whole‐cell protein, cells at 70%–80% confluence were lysed in ice‐cold 1× RIPA buffer (9806S; Cell Signaling Technology) containing 1 mm PMSF. The protein concentration was measured using a BCA protein assay (23225; Thermo Fisher Scientific) according to the manufacturer's instructions. Equal amounts of proteins were subjected to SDS‒polyacrylamide gel electrophoresis and transferred to PVDF membranes (IPVH00010, Millipore). The membranes were then blocked in 5% nonfat milk for 60 min and incubated with a primary antibody at 4°C overnight. The membranes were subsequently incubated with the corresponding HRP‐conjugated secondary antibody for 2 h at room temperature. Protein detection was performed using Clarity Western ECL Substrate (1705060; Bio‐Rad) on a ChemiDoc MP Imaging System (Bio‐Rad).

For the DLAT oligomerization assay, cells were lysed in ice‑cold 1× RIPA buffer (9806S; Cell Signaling Technology) containing 1 mm PMSF and supplemented with Benzonase. After centrifugation at 12 000 g for 10 min at 4°C, the lysates were mixed with 5× denaturing and non‑reducing gel sample loading buffer, heated at 70°C for 10 min, and then subjected to immunoblot analysis.

### 2.9 Immunofluorescence

Cells seeded onto sterile glass coverslips were fixed with 4% paraformaldehyde at room temperature for 10 min, permeabilized with 0.2% Triton X‐100, blocked with 4% BSA at room temperature, and incubated with primary antibodies at 4°C overnight. The cells were then incubated with a secondary antibody at room temperature for 1 h and stained with 1 µg/ml DAPI (Sigma, D9542) for 5 min. For standard confocal immunofluorescence imaging, cells were imaged using an LSM980 microscope (Carl Zeiss). Total internal reflection fluorescence (TIRF) data were collected using a Polar‑SIM system (Airy Technologies Co., Ltd., China). All acquired images were processed and analyzed with ImageJ software (version 1.54).

### 2.10 Cell Viability Assays

Cells were seeded in a 96‐well plate with 3 replicate wells per group and incubated overnight at 37°C in a cell culture incubator. Following overnight incubation, the cells were treated with the indicated drugs for 72 h. Then, 10 µl of MTT (final concentration of 0.5 mg/mL) was added to each well, and the cells were incubated at 37°C for 4 h. After the supernatant was removed, the formazan crystals were dissolved in 200 µl of DMSO. The absorbance at 490 nm was measured using a microplate reader (Epoch Biotek, USA) to assess cell viability.

### 2.11 Flow Cytometry

To assess the degree of cell death under different treatments, an Annexin V‐FITC/PI double‐staining apoptosis detection kit (KGA1102‐100) was used according to the manufacturer's instructions. Cells in the logarithmic growth phase were subjected to the indicated treatments, collected in a centrifuge tube, and washed twice with ice‐cold PBS. The cell pellet was subsequently resuspended in 500 µL of binding buffer. Next, 5 µL of propidium iodide was added, followed by mixing; the mixture was allowed to react for 10 min at room temperature in the dark. The stained cells were analyzed using CytExpert software on a cytoFLEX S (Beckman Coulter).

### 2.12 Radiation and Clonogenic Survival Assays

For all clonogenic survival assays, irradiation was conducted with an RS2000 biological irradiator at the indicated dose. To determine the effects of different treatments, 1000 cells per well were seeded in triplicate in 6‐well plates and incubated overnight. The cells were then pretreated with different treatments and then irradiated. Fresh medium containing different drugs was added to the cells every 3 days. After incubation for 1–2 weeks, the cells were stained with 0.5% crystal violet (C0775; Sigma) dissolved in 20% methanol. The colonies in each well were counted visually, and the survival fraction was calculated using GraphPad Prism 8 and normalized to that of unirradiated control cells.

### 2.13 Quantitative Real‐Time PCR

RNA was isolated from cultured cells via an EZ‐press RNA Purification Kit (EZBioscience, B0004D). The conversion of RNA to cDNA was accomplished using a PrimeScript RT Reagent Kit (Takara, RR047B). Subsequently, quantitative PCR was performed with SYBR Premix Ex Taq II (Takara, RR820B) on a Light Cycler 480 system (Roche Diagnostics) according to the manufacturer's instructions. All genes were examined in triplicate.

The primers used to amplify PRMT5 were 5’‐CGATCAGACCTACTGCTGTCA‐3’ (forward) and 5’‐CTCGGAGTTCCTGCGAATCT‐3’ (reverse); the primers used to amplify VPS34 were 5’‐GCTGTGCTGGATATTGCGTG‐3’ (forward) and 5’‐GGTGGAGGAAAGCCGTGTAA‐3’ (reverse); and the primers used to amplify GAPDH were 5’‐AAGCCTGCCGGTGACTAAC‐3’ (forward) and 5’‐GTTAAAAGCAGCCCTGGTGAC‐3’ (reverse).

### 2.14 Proteomic Analysis

To detect changes in protein levels within cancer cells during the induction of cuproptosis, SBC‐2 cells were treated with 40 nm elesclomol (with 1 µm CuCl2 in the media) for 48 h and collected for data‐independent acquisition (DIA) quantitative proteomics analysis. Each sample was treated with SDT lysis buffer, incubated in a boiling water bath for 3 min, subjected to ultrasonic disruption for 2 min, and centrifuged at 16 000 × g for 20 min at 4°C. The supernatant was collected, and the protein concentration was quantified using the BCA method. The protein from each sample was digested using the filter‐aided sample preparation (FASP) method, dried, reconstituted with 0.1% formic acid (FA), and measured via LC‒MS analysis. The peptides were separated using a Vanquish Neo UHPLC system (Thermo Scientific) and analyzed via DIA mass spectrometry on an Orbitrap Astral mass spectrometer (Thermo Scientific). The data were merged and analyzed using DIA‐NN software with the UniProt‐*Homo sapiens* (Human) [9606]‐207981‐20230717.fasta database and a q value ≤0.01 as the selection parameter.

For protein interaction analysis with VPS34, affinity‐isolated VPS34 protein was digested using FASP, reconstituted in Nano‐LC mobile phase A (0.1% formic acid/water), and analyzed via LC‒MS in Data Dependent Analysis (DDA) mode. MS/MS data were analyzed using PEAKS Studio 8.5 with a 1.0% local false discovery rate at the PSM after searching the *Homo sapiens* database, with precursor and fragment mass tolerances set to 10 ppm and 0.05 Da, respectively, and modifications, including oxidation (M), acetylation (protein N‐term), deamidation (NQ), Pyro‐glu from E, Pyro‐glu from Q, and carbamidomethylation of cysteine.

For posttranslational modification analysis, affinity‐isolated VPS34 protein was electrophoresed on SDS‒polyacrylamide gels, excised, washed, destained, treated with ammonium bicarbonate and acetonitrile, and digested overnight with trypsin. The peptides were treated with extraction buffer, sonicated, concentrated, reconstituted in Nano‐LC mobile phase A, and analyzed by LC‒MS in DDA mode, with the MS‒MS data processed like those used for the protein interaction analysis.

### 2.15 Immunoprecipitation (IP)

IP extracts were prepared using RIPA buffer supplemented with 1 mm PMSF. The extracts were then incubated with specific antibodies at 4°C overnight on a rotating platform. Next, protein A/G magnetic beads (B23201, Selleck) were added to the mixtures, which were subsequently incubated at 4°C for 2 h on a rotator. Next, the beads were washed 4 times in ice‐cold RIPA lysis buffer and subsequently boiled in 2× SDS‒PAGE buffer. The supernatant was subjected to Western blotting or MS to analyze the coprecipitated proteins or modification of the target protein.

### 2.16 Molecular Docking

To construct a complex model of PRMT5 and VPS34, we performed docking simulations of PRMT5 with VPS34. The docking simulation was performed in HDOCK server 1 (http://hdock.phys.hust.edu.cn/). The crystal structure of PRMT5 was downloaded from the RCSB Protein Data Bank (https://www.rcsb.org/) under accession entry 7L1G. The structure of VPS34 was constructed on the basis of the crystal structure of VPS34 (PDB entry 7BL1), and the missing residues of VPS34 were built with the help of Loop Modeler in MOE software (Molecular Operating Environment software, Chemical Computing Group ULC, version 2022.02). For protein‒protein docking of PRMT5 and VPS34, PRMT5 was set as the receptor, while VPS34 was set as the ligand. The HDOCK server automatically predicted their interaction through a hybrid algorithm of template‐based and template‐free docking. The interactions between the two proteins in the PRMT5‐VPS34 complex were further analyzed via Molecular Operating Environment software (MOE; Chemical Computing Group ULC, version 2022.02) and illustrated via PyMOL (Schrödinger, LLC, version 2.5.0).

### 2.17 Subcutaneous Xenograft Model

Female BALB/c nude mice were purchased from GemPharmatech Co., Ltd. (Guangdong, China) and were 6–7 weeks old. All the mice were maintained under specific pathogen‐free conditions with a 12‐h light/dark cycle. SBC‐2 cells were subcutaneously injected into the right axilla of the mice (5 × 10^6^ cells/mouse). After 5 days, the mice were randomly divided into different treatment groups. The tumors were irradiated with an RS2000 biological irradiator at a dose of 4 Gy. Elesclomol was administered intraperitoneally at 100 mg/kg every 2 days. PRMT5 and VPS34 inhibitors were given by gavage at 100 mg/kg daily. These treatments were administered twice before irradiation and then every 2 days or daily until the study endpoint, as depicted in the figures. The tumor volume was measured 3 or 4 times per week until the endpoint and calculated using the following formula: 0.5 × length × width^2^. After the mice were euthanized, the tumor tissues were excised and weighed. All animal care and experimental procedures were approved by the Institutional Animal Care and Use Committee (IACUC) of Sun Yat‐sen University Cancer Center (Approval No. L025501202308014).

### 2.18 Analyses of Public Data

The AUC values for elesclomol and proteomic data of pancancer cell lines were obtained from the Cancer Cell Line Encyclopedia (CCLE, https://depmap.org/portal/). Correlation analysis between protein expression and AUC values was conducted using R software. Data for evaluating the prognostic value of VPS34 and PRMT5 protein expression were acquired from the Gene Expression Omnibus (GSE32649877), while data concerning the relationship between the expression of PRMT5 and radiotherapy sensitivity were acquired from The Cancer Genome Atlas (TCGA, https://portal.gdc.cancer.gov/). Among patients with 33 different cancer types, those who had undergone radiotherapy were included in the analysis. Those with a complete response (CR) or partial response (PR) were deemed radiotherapy sensitive (RS), whereas those with progressive disease (PD) or stable disease (SD) were deemed radiotherapy resistant (RR). Survival analysis was conducted with the R packages “survival” and “survminer.”

### 2.19 Statistical Analysis

GraphPad Prism 8 and R software (4.3.2) were used for the statistical analyses. The results are expressed as the means ± SDs from at least three biologically independent experiments or samples. The data were analyzed using unpaired Student's t test, one‐way or two‐way ANOVA with Dunnett's or Tukey's multiple comparisons test, or the Wilcoxon matched‐pairs signed‐rank test, which were conducted with GraphPad Prism. Statistical tests were two‐sided, and significance was defined as a *p* value less than 0.05.

## 3 Results

### 3.1 Genome‐Wide CRISPRa Screening Identified VPS34 as a Negative Regulator of Cuproptosis

To systematically identify host factors that confer resistance to copper toxicity, we performed a genome‐wide CRISPR activation (CRISPRa) screen in cells challenged with the copper ionophore elesclomol‐copper (ES‐Cu) (Figure 1A). The fidelity of our screen was validated by the distribution of known regulators in the RRA score‐rank plot: copper exporters and chelators (e.g., ATP7A, MT2A, and MT2B) were enriched among the top positive hits, whereas key cuproptosis effectors (e.g., FDX1 and DLAT) and the importer SLC31A1 ranked prominently as negative hits (Figure 1B). Functional enrichment analysis of the top 500 significant hits revealed a striking overrepresentation of pathways associated with macroautophagy and the vacuolar membrane (Figure 1C). Detailed network mapping further revealed a cluster of enriched genes involved in autophagy and the endosome membrane trafficking system (Figure 1D,E).

**FIGURE 1:**
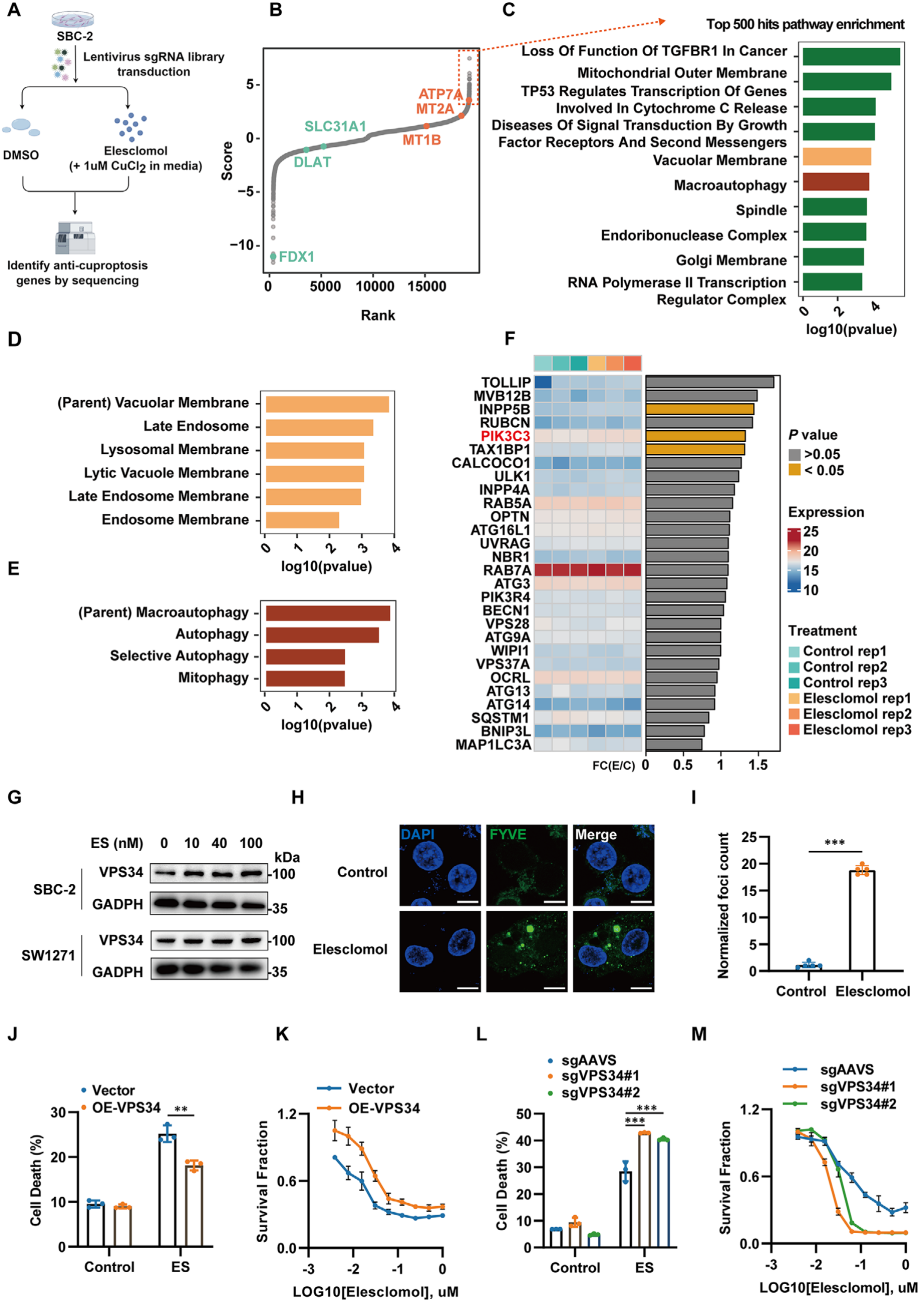
Genome‐wide CRISPRa screening identified VPS34 as a negative regulator of cuproptosis. (A) Schematic illustration of the genome‐wide CRISPR activation (CRISPRa) screening strategy used to identify genes conferring resistance to elesclomol‐Copper (ES‐Cu)‐induced cuproptosis. Cells were collected from the surviving population of elesclomol‐Cu‐treated SBC‐2 cells transduced with a genome‐wide lentiviral sgRNA library. Genomic DNA was subsequently isolated, and the genes related to cuproptosis resistance were identified by sequencing. (B) Rank plot of the robust rank aggregation (RRA) scores for all the genes. Key cuproptosis‐positive regulators and negative regulators are highlighted. (C) Pathway analysis of genes from the top 500 significant hits. Gene Ontology analysis was performed using Metascape. (D) A subset of enriched GO terms related to the vacuolar membrane pathway ranked on the basis of their significance (‐log10 p value). (E) A subset of enriched GO terms related to the macroautophagy pathway ranked on the basis of their significance (‐log10 p value). (F) Heatmap of the protein expression of autophagy endosome‐related genes in SBC‐2 cells treated with 40 nm elesclomol for 48 h (+1 µm CuCl2 in media). (G) Protein content in SBC‐2 cells treated with the indicated concentrations of elesclomol for 48 h (+1 µm CuCl2 in media). (H) Confocal immunofluorescence imaging of SBC‐2 cells pretreated with DMSO or 40 nm elesclomol in the presence of 1 µm CuCl2 in the medium (FYVE‐green, DAPI‐blue). (I) Foci were segmented and quantified in each condition. Error bars are the means ± SDs; n = 3 independent repeats. *P* values were calculated using two‐tailed unpaired Student's t tests (^**^ ^*^p < 0.001). (J) Levels of cell death in the indicated cells treated with 40 nm elesclomol in the presence of 1 µm CuCl2 in the medium for 72 h. The error bars represent the means ± SDs; n = 3 independent experiments. *P* values were calculated using two‐tailed unpaired Student's t tests (^*^ ^*^p < 0.01). (K) Viability of SBC‐2 cells overexpressing VPS34 following treatment with elesclomol in the presence of 1 µm CuCl2 in the medium. (L) Levels of cell death among the indicated cells treated with 40 nm elesclomol in the presence of 1 µm CuCl2 in the medium for 72 h. The error bars represent the means ± SDs; n = 3 independent experiments. *P* values were calculated using one‐way ANOVA with Dunnett's multiple comparisons test (^*^ ^*^p < 0.01, ^**^ ^*^p < 0.001). (M) Viability of SBC‐2 cells with CRISPR/Cas9‐mediated deletion of VPS34 following treatment with elesclomol in the presence of 1 µm CuCl2 in the medium. (J, L) The error bars represent the means ± SDs; n = 3 independent experiments. P values were calculated using two‐tailed unpaired Student's t tests (^*^ ^*^p < 0.01). All the original blots can be found in Supporting File S2.

Prompted by these screening results, we sought to validate the functional relevance of autophagy in the cellular response to copper stress. ES‐Cu treatment triggered robust autophagy activation, as evidenced by the accumulation of LC3‐II, degradation of p62, and formation of LC3 puncta (Figure S1A–C). Importantly, pharmacological modulation confirmed that this autophagic response is protective: inhibiting autophagy with chloroquine (CQ) exacerbated intracellular copper accumulation and sensitized cells to death, whereas activating autophagy with AZD8055 reduced copper levels and mitigated toxicity (Figure S1D–F). These data confirm that autophagy is a bona fide defense mechanism against cuproptosis.

To identify the specific molecular effector driving this protective response, we performed DIA proteomic profiling to analyze protein expression changes within the enriched pathways upon copper stimulation. Among all the candidates, VPS34 (PIK3C3), the catalytic subunit of class III PI3K, exhibited the most significant upregulation according to the heatmap (Figure 1F). Notably, VPS34 functions as a crucial convergence point for these enriched pathways, generating phosphatidylinositol 3‐phosphate (PI3P) to orchestrate both endocytic trafficking and autophagosome formation. This induction was further corroborated by Western blotting, which revealed a dose‐dependent increase in VPS34 expression upon ES‐Cu treatment (Figure 1G). Consistent with its increased expression, intracellular VPS34 enzymatic activity was elevated, as indicated by the increase in FYVE‐positive PI3P puncta following copper stress (Figure 1H,I). We further verified the functional necessity of VPS34. The overexpression of VPS34 significantly promoted resistance to cuproptosis (Figure 1J,K), whereas its depletion via sgRNA markedly sensitized cells to ES‐Cu treatment (Figure 1L,M; Figure S1G–J). Moreover, our assessment of DLAT oligomerization revealed that the loss of VPS34 significantly exacerbated DLAT oligomerization during cuproptosis, which could be blocked by siRNA‐mediated silencing of FDX1 (Figure S1K,L). Collectively, our screening and validation strategies identify VPS34 as a critical, stress‐inducible guardian against cuproptosis.

### 3.2 VPS34 Maintains Intracellular Copper Homeostasis by Coordinating SLC31A1 and ATP7A Trafficking

Since cuproptosis is fundamentally driven by the accumulation of intracellular free copper, limiting copper overload is a prerequisite for cell survival. To determine whether VPS34 confers resistance by modulating cellular copper content, we analyzed intracellular copper dynamics. Flow cytometry analysis revealed that overexpression of VPS34 significantly reduced intracellular copper levels (Figure S2A,B). These findings were visually corroborated by confocal microscopy using the specific copper fluorescent probe Coppersensor‐1 (CS1), which resulted in a marked decrease in copper signals in the VPS34‐overexpressing cells (Figure S2C). Conversely, genetic ablation of VPS34 resulted in substantial accumulation of intracellular copper, as evidenced by both flow cytometry (Figure 2A,B) and CS1 probe imaging (Figure 2C). These data establish that VPS34 activity is necessary to prevent lethal copper accumulation.

**FIGURE 2:**
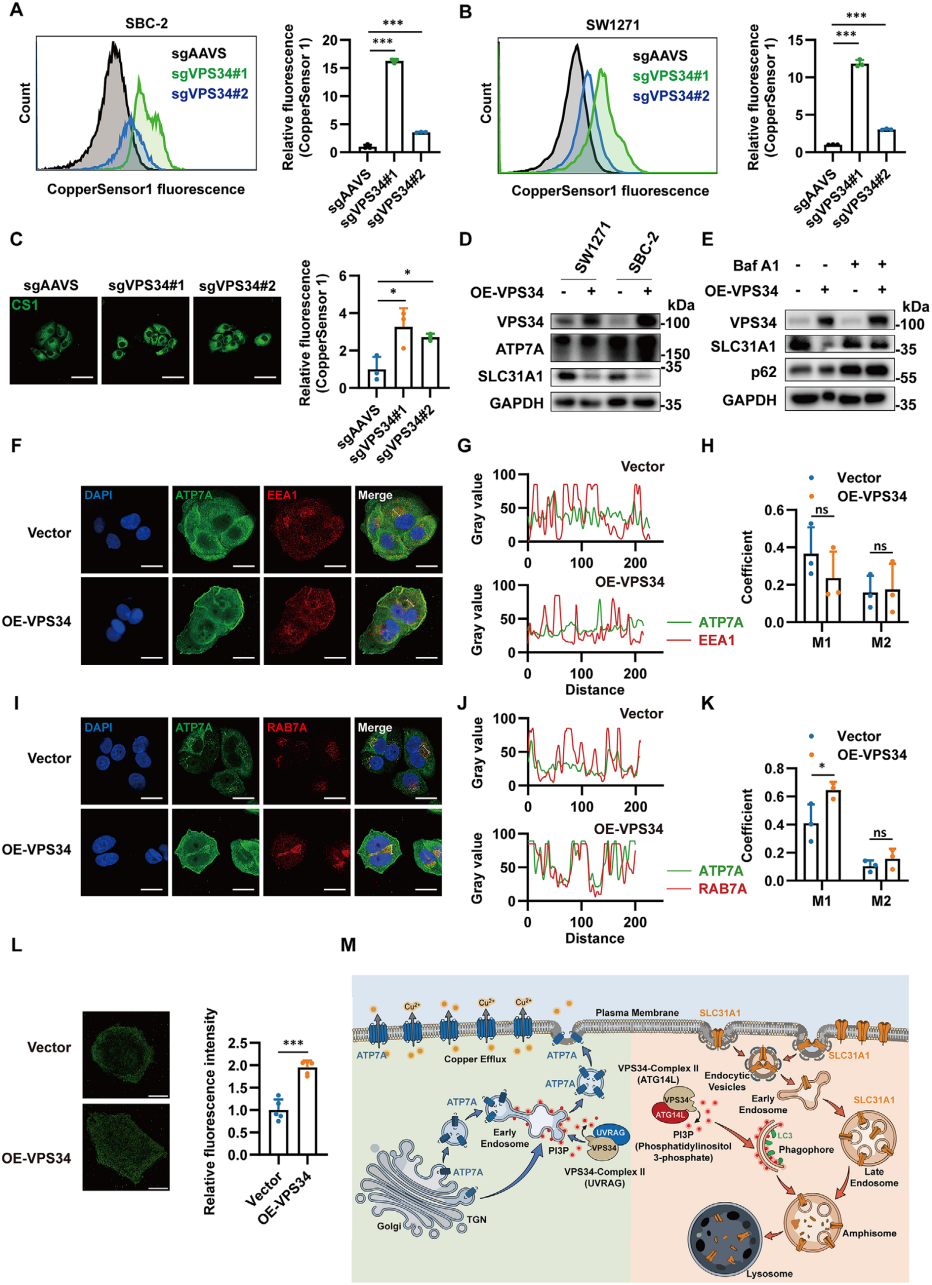
VPS34 maintains intracellular copper homeostasis by coordinating SLC31A1 degradation and ATP7A trafficking. (A,B) Copper levels detection of CopperSensor 1 (CS1) in SBC‐2 and SW1271 cells with CRISPR/Cas9‐mediated deletion of VPS34 following treatment with 1 µm CuCl2 in the medium. (C) Copper ions were analyzed with CS1 in the indicated SBC‐2 cells following treatment with 1 µm CuCl2 in the medium. *n* = 3 biologically independent experiments. Scale bars, 50 µm. (A–C) *p* values were calculated using one‐way ANOVA with Dunnett's multiple comparisons test (* p < 0.05, *** p < 0.001). (D) Western blot showing the expression of copper transporters in the indicated cells expressing empty vector or with VPS34 overexpression. (E) Western blot showing the expression of copper transporters in the indicated cells expressing empty vector or with VPS34 overexpression following treatment with 500 nm Baf A1 for 12 h. (D,E) Data are representative of *n* = 3 biologically independent experiments. (F,I) Representative confocal immunofluorescence images showing the subcellular distribution of ATP7A (green) and EEA1 or RAB7A (red) in control and VPS34‐overexpressing cells. Scale bars, 10 µm (G–J) Colocalization analysis of ATP7A with the endosome marker EEA1 or RAB7A using ImageJ. (H,K) Quantitative colocalization analysis using Manders’ Colocalization Coefficients (MCC). Specifically, M1 represents the fraction of red signal (EEA1, RAB7A, etc.) overlapping with green (ATP7A), while M2 represents the fraction of green overlapping with red. *n* = 3 biologically independent experiments. (L) Representative Total Internal Reflection Fluorescence (TIRF) microscopy images (left) and quantitative analysis of relative fluorescence intensity (right) of ATP7A in SBC‐2 cells expressing empty vector or OE‐VPS34. Scale bars, 10 µm. Data are presented as mean ± SDs. (H,K,L) ^*^p < 0.05, ^**^ ^*^p < 0.001, *ns*: *p* > 0.05, as determined by unpaired two‐tailed Student's t‐test. (M) Schematic model depicting the bimodal regulation of copper homeostasis by VPS34: promoting the lysosomal degradation of SLC31A1 and facilitating the RAB7A‐dependent recycling of ATP7A. All the original blots can be found in Supporting File S2.

To investigate the molecular basis of this copper reduction, we profiled the key transporters governing copper homeostasis: the influx transporter SLC31A1 and the efflux transporters ATP7A and ATP7B. Given that ATP7B expression is largely restricted to hepatic tissues, we focused our analysis on SLC31A1 and ATP7A in our lung cancer models. Western blotting revealed a distinct regulatory pattern: while the total protein abundance of ATP7A remained unchanged, overexpression of VPS34 led to a significant reduction in SLC31A1 levels (Figure 2D). Given that SLC31A1 is known to undergo autophagy‐lysosomal degradation in response to copper stress, we hypothesized that VPS34 might accelerate this specific turnover pathway. To test this hypothesis, we treated cells with the lysosomal inhibitor BafA1. Indeed, lysosomal inhibition successfully prevented the VPS34‐induced reduction in SLC31A1 (Figure 2E), confirming that VPS34 limits copper influx by facilitating the lysosomal degradation of SLC31A1.

We next investigated the fate of ATP7A. Since its total protein abundance remained stable, we hypothesized that VPS34 modulates its subcellular distribution. Confocal microscopy revealed that upon VPS34 overexpression, ATP7A was strikingly relocalized to the plasma membrane (Figure 2F–I,L). To map the specific intracellular trafficking route, we performed colocalization assays with a panel of organelle markers, including EEA1 (early endosome), RAB7A (late endosome), LAMP1 (lysosome), and LC3 (autophagosome). Notably, while ATP7A showed minimal overlap with EEA1, LAMP1, or LC3 (Figure 2F–H; Figure S2D–G), VPS34 overexpression significantly enhanced its colocalization specifically with RAB7A (Figure 2I–K). These data indicate that VPS34 facilitates the sorting of ATP7A to the cell surface via a RAB7A‐positive endosomal pathway, effectively bypassing degradative compartments.

Collectively, these findings delineate a dual‐mechanism model for VPS34‐mediated copper homeostasis (Figure 2M): on the one hand, VPS34 restricts copper influx by promoting the degradation of SLC31A1; on the other hand, it enhances copper efflux by facilitating the sorting of ATP7A from RAB7A‐positive endosomes to the plasma membrane.

### 3.3 PRMT5 Interacts with VPS34 and Mediates Its Symmetric Dimethylation at Arginine 174

To identify factors that regulate the stability of the VPS34 protein, we performed high‐throughput mass spectrometry followed by immunoprecipitation and identified 694 proteins that interact with VPS34. We then performed correlation analysis on the CCLE database to identify proteins whose expression was moderately or strongly correlated with elesclomol sensitivity. By integrating the above two methods, we determined that PRMT5 interacts with VPS34 and plays a significant role in regulating cuproptosis sensitivity, making it an ideal druggable target for treating radioresistant cancers (Figure 3A). PRMT5 is a symmetric arginine methyltransferase that is closely associated with radioresistance, and PRMT5 inhibitors have entered clinical trials as promising anticancer agents [12, 15]. Using qPCR and Western blot analyses, we revealed that both the mRNA and protein expression of PRMT5 increased in a dose‐dependent manner after treatment with elesclomol (Figure 3B,C). On the basis of the aforementioned results, it is plausible that PRMT5 plays a critical role in regulating VPS34 stability and safeguarding cells against cuproptosis. To confirm the interaction between PRMT5 and VPS34, coimmunoprecipitation assays were performed. As shown in HEK293T cells, PRMT5 robustly interacted with VPS34 at both the endogenous and exogenous levels (Figure 3D,E). VPS34 is composed of three domains: the C2 domain, the PIK domain, and the kinase domain. The C2 domain plays a vital role in VPS34 complexes I and II by establishing crucial interactions with other core subunits. To precisely identify the VPS34 domain that interacts with PRMT5, we created a series of Flag‐tagged deletion mutants. Immunoprecipitation assays confirmed that the PI3K C2 domain of VPS34 directly binds to PRMT5 (Figure 3F). Next, we further investigated the regulation of VPS34 arginine methylation by PRMT5 by immunoprecipitation with an antibody against symmetric dimethylated arginine (SDMA). The results revealed that VPS34 has symmetric arginine methylation and that the overexpression of PRMT5 led to an elevated level of VPS34 SDMA modification. Conversely, knockdown of PRMT5 by siRNA markedly reduced the level of SDMA (Figure 3G,H). These experiments demonstrated that PRMT5 can regulate VPS34 by interacting with it and mediating its symmetric dimethylation. To pinpoint the SDMA modification site on VPS34, we performed a mass spectrometry assay and detected R174 as a dimethylated arginine residue (Figure 3I,J). Moreover, R174 of human VPS34 is phylogenetically conserved across vertebrates, suggesting that this methylation may have important biological functions. To further validate the methylation of VPS34 at R174, we expressed a methylation inactivation mutant variant (R174K). Compared with WT VPS34, the R174K mutant exhibited reduced methylation (Figure 3K). Additionally, an antibody specific for the VPS34 R174 SDMA modification was generated (anti‐VPS34‐R174me2s). Western blot analysis of SBC‐2 cells revealed that PRMT5 overexpression significantly increased VPS34 methylation at R174, whereas PRMT5 knockdown markedly reduced R174 methylation when this specific antibody was used (Figure 3L,M). To further validate our findings concerning the PRMT5‐VPS34 interaction, molecular docking was conducted. The model showed that the Arg174 acceptor site of VPS34 could dock into the active site of PRMT5, with the distance between the methyl group of SAM and the guanidyl group of Arg174 being approximately 5 Å, which is suitable for a reaction. Residues such as Q171, R174, M187, K177, K132, and Y133 in VPS34 bind to PRMT5 through hydrogen bonds and salt bridge interactions involving residues such as E325, E328, N396, V399, N403, F406, and E407 (Figure 3N,O). These interactions are the main interactions that contribute to the binding affinity between VPS34 and PRMT5. Collectively, these findings suggest that PRMT5 acts as a methyltransferase responsible for VPS34 methylation at R174.

**FIGURE 3:**
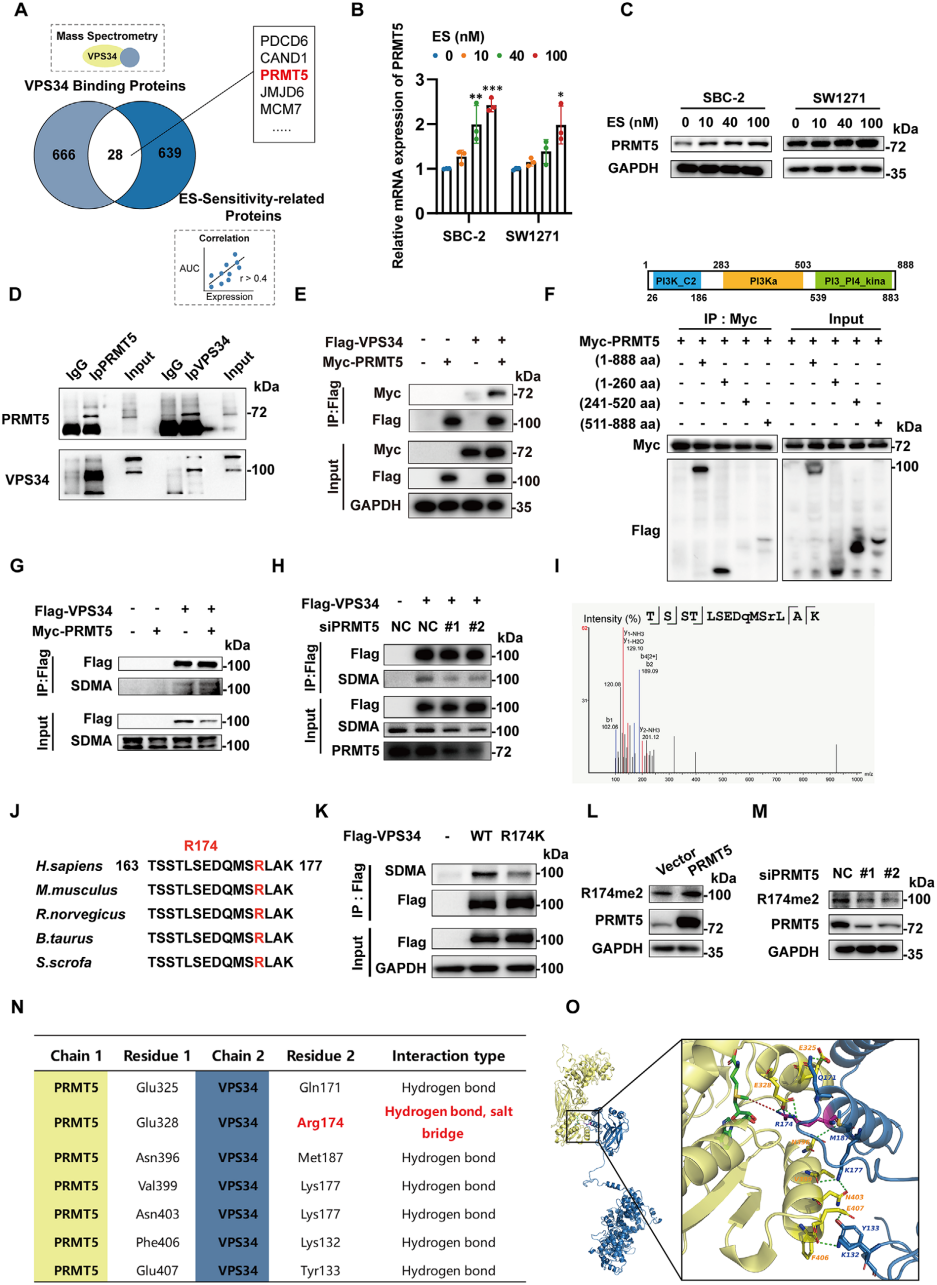
PRMT5 catalyzes the SDMA of VPS34 at R174. (A) Venn diagram illustrating the overlap between VPS34‐binding proteins and elesclomol‐sensitivity‐related proteins. (B) Relative mRNA expression of PRMT5 in the indicated cells following treatment with elesclomol in the presence of 1 µm CuCl2 in the medium. The error bars represent the means ± SDs; n = 3 independent repeats. P values were calculated using two‐tailed unpaired Student's t test (^*^p < 0.05, ^*^ ^*^p < 0.01, ^**^ ^*^p < 0.001). (C) Western blot showing the expression of PRTM5 in the indicated cells following treatment with elesclomol for 48 h in the presence of 1 µm CuCl2 in the medium. (D) Validation of the PRMT5‐VPS34 interaction via immunoprecipitation on the basis of endogenous expression and immunoprecipitation of the indicated proteins. Normal IgG was used as the negative control. (E) Validation of the PRMT5‐VPS34 interaction via immunoprecipitation on the basis of exogenous expression and immunoprecipitation of the indicated proteins with different tags. (F) Identification of the VPS34 region that interacts with PRMT5. (G,H) The SDMA levels of VPS34 were determined by immunoprecipitation. (I) Mass spectrum of the SDMA modification at R174 of VPS34. (J) Alignment of the consensus VPS34 amino acid sequences around the arginine 174 residue (highlighted in red) among various species. (K) The SDMA levels of VPS34 were determined by immunoprecipitation. (L, M) The SDMA modification level of R174 in VPS34 was determined by Western blot analysis using an R174me2s site‐specific antibody. (N) Contact list between PRMT5 and VPS34. (O) Predicted binding model of PRMT5 and VPS34. PRMT5 is shown in pale yellow, whereas VPS is blue. The SAM in PRMT5 is shown as a green stick. The interacting residues in PRMT5 and VPS34 are depicted as yellow and blue sticks, respectively. R174 in VPS34 is shown as a magenta stick. The hydrogen bond and salt bridge interactions between PRMT5 and vps34 are depicted as green and blue dashed lines, respectively. All western blot data are representative of *n* = 3 biologically independent experiments. All the original blots can be found in Supporting File S2.

### 3.4 PRMT5 Stabilizes VPS34 by Recruiting USP10 to Prevent K48‐Linked Polyubiquitination

To elucidate the mechanism by which PRMT5 regulates VPS34 abundance, we first examined the effect of PRMT5 on VPS34 stability. We observed that while PRMT5 overexpression significantly increased VPS34 protein levels, it did not affect VPS34 mRNA expression; conversely, PRMT5 knockdown reduced VPS34 protein levels, suggesting a posttranslational regulatory mechanism (Figure 4A–D). To determine the degradation pathway involved, we treated cells with specific inhibitors. The proteasome inhibitor MG132, but not the lysosome inhibitor bafilomycin A1, significantly extended the half‐life of VPS34, indicating that VPS34 undergoes turnover via the ubiquitin‒proteasome system (UPS) (Figure 4E,F). This finding was further confirmed by cycloheximide (CHX) chase assays: PRMT5 overexpression prolonged the half‐life of VPS34, whereas PRMT5 depletion or the methylation‐deficient R174K mutation accelerated its degradation (Figure 4G–I).

**FIGURE 4:**
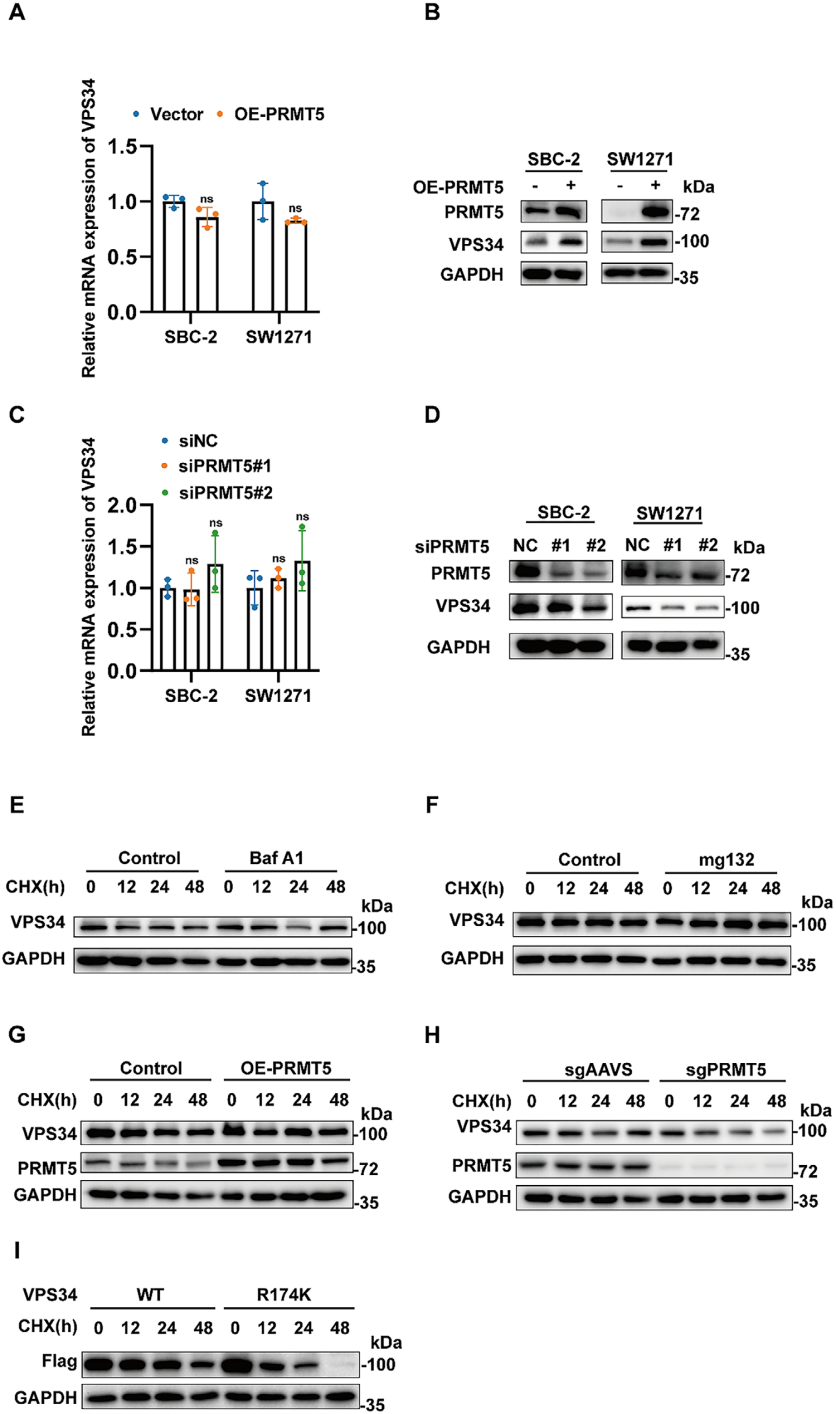
SDMA on VPS34 blocks VPS34 degradation and promotes autophagy. (A) Relative mRNA expression of VPS34 in the indicated cells following siRNA‐mediated PRMT5 knockdown. The error bars represent the means ± SDs; n = 3 independent repeats. P values were calculated using two‐tailed unpaired Student's t tests (ns, p > 0.05). (B) Western blot showing the expression of VPS34 in the indicated cells following siRNA‐mediated PRMT5 knockdown. (C) Relative mRNA expression of VPS34 in the indicated cells overexpressing PRMT5. The error bars represent the means ± SDs; n = 3 independent repeats. P values were calculated using two‐tailed unpaired Student's t tests (ns, *p* > 0.05). (D) Western blot showing the expression of VPS34 in the indicated cells overexpressing PRMT5. (E) Western blot showing the expression of VPS34 in SBC‐2 cells treated with DMSO or 100 nm Baf A1 for 12 h, followed by treatment with 10 µg/ml CHX for the indicated times. (F) Western blot showing the expression of VPS34 in SBC‐2 cells treated with DMSO or 10 µm mg132 for 12 h, followed by treatment with 10 µg/ml CHX for the indicated times. (G) Western blot showing the expression of VPS34 in SBC‐2 cells overexpressing PRMT5, followed by treatment with 10 µg/ml CHX for the indicated times. (H) Western blot showing the expression of VPS34 in SBC‐2 cells following CRISPR/Cas9‐mediated deletion of PRMT5 followed by treatment with 10 µg/ml CHX for the indicated times. (I) Western blot showing the expression of VPS34 in SBC‐2 cells overexpressing wild‐type VPS34 (WT) or R174K VPS34 (R174K) followed by treatment with 10 µg/ml CHX for the indicated times. All western blot data are representative of *n* = 3 biologically independent experiments. All the original blots can be found in Supporting File S2.

Since ubiquitination is the prerequisite signal for proteasomal degradation, we hypothesized that PRMT5‐mediated methylation antagonizes VPS34 ubiquitination. Indeed, immunoprecipitation assays revealed that PRMT5 overexpression significantly suppressed VPS34 ubiquitination, whereas PRMT5 knockdown or MG132 treatment led to the accumulation of polyubiquitinated VPS34 species (Figure 5A,C). Using linkage‐specific antibodies, we determined that VPS34 is primarily modified by K48‐linked rather than K63‐linked ubiquitin chains. Importantly, the PRMT5 level was negatively correlated with the intensity of K48‐linked ubiquitination of VPS34 (Figure 5D,E). Furthermore, compared with the wild‐type protein, the methylation‐deficient R174K mutant exhibited hyperubiquitination (Figure 5F), confirming that methylation at R174 acts as a brake on K48‐linked ubiquitination.

**FIGURE 5:**
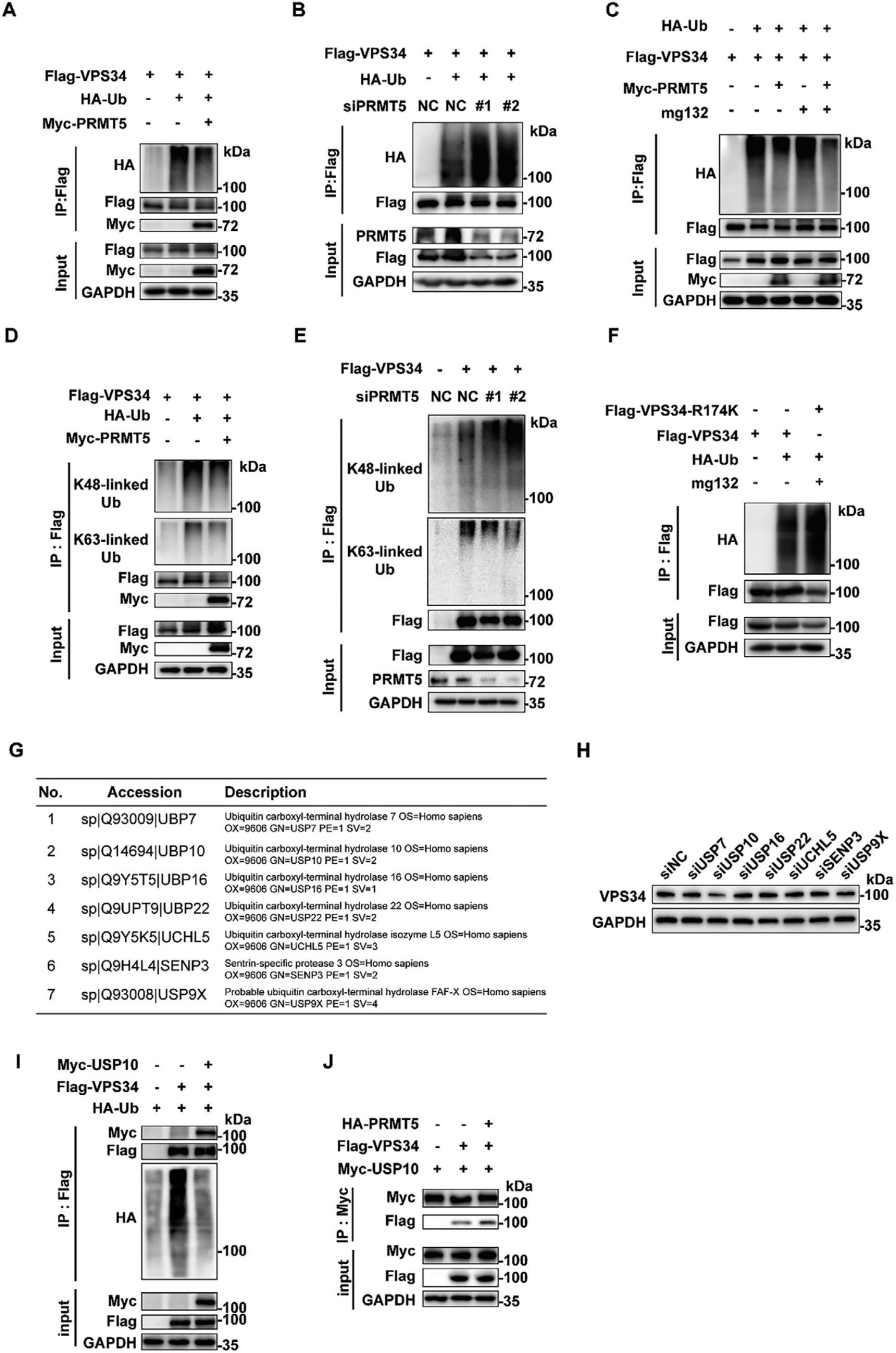
USP10‐mediated deubiquitination stabilizes VPS34. (A) Immunoprecipitation results showing that PRMT5 overexpression decreased VPS34 ubiquitination. (B) Immunoprecipitation results showing that PRMT5 knockdown by siRNA increased VPS34 ubiquitination. (C) Immunoprecipitation results showing that treatment with MG132 led to the accumulation of ubiquitinated VPS34. (D, E) Immunoprecipitation results showing that the ubiquitination of VPS34 is K48‐linked. (F) Immunoprecipitation results showing that VPS34‐R174K increased VPS34 ubiquitination. (A–F) HEK293T cells were transfected with the indicated plasmids, and immunoprecipitation with anti‐Flag affinity gels and immunoblotting were performed. (G) Candidate deubiquitinating enzymes identified via mass spectrometry. (H) Western blot showing the expression of VPS34 in the indicated cells following siRNA‐mediated knockdown of the indicated genes. (I) Immunoprecipitation results showing that USP10 overexpression decreased VPS34 ubiquitination. HEK293T cells were transfected with the indicated plasmids, and immunoprecipitation with anti‐Flag affinity gels and immunoblotting were performed. (J) Immunoprecipitation results showing that PRMT5 overexpression decreased the interaction between VPS34 and USP10. HEK293T cells were transfected with the indicated plasmids, and immunoprecipitation with anti‐Myc affinity gels and immunoblotting were performed. All western blot data are representative of *n* = 3 biologically independent experiments. All the original blots can be found in Supporting File S2.

To understand the specific mechanism by which SDMA mediates VPS34 deubiquitination, we identified and analyzed seven deubiquitinating enzymes that bind to VPS34 through MS (Figure 5G). By knocking down these enzymes in SBC‐2 cells with siRNA, we discovered that USP10 knockdown significantly reduced VPS34 protein levels, suggesting that USP10 may be involved in VPS34 deubiquitination (Figure 5H). To confirm these results, we conducted coimmunoprecipitation assays and confirmed the robust physical interaction between VPS34 and USP10. Crucially, we found that this recruitment is methylation dependent: PRMT5 overexpression significantly increased the binding affinity between VPS34 and USP10 (Figure 5I,J). Collectively, these data demonstrate that PRMT5‐mediated R174 methylation serves as a molecular scaffold to recruit USP10, which antagonizes K48‐linked polyubiquitination to stabilize VPS34.

### 3.5 Methylation at Arg174 Is Essential for VPS34 Stability and Cellular Resistance to Cuproptosis

Having established that PRMT5 stabilizes VPS34, we reasoned that PRMT5 should functionally mirror VPS34 in regulating copper homeostasis and cell survival. To test this hypothesis, we modulated PRMT5 expression and monitored intracellular copper dynamics. Flow cytometry and confocal microscopy with a CS1 probe revealed that PRMT5 overexpression significantly reduced the intracellular copper concentration (Figure S3A–C). Conversely, PRMT5 knockout (KO) led to marked accumulation of copper (Figure 6A–C). Mechanistically, consistent with our findings on VPS34, PRMT5 overexpression downregulated SLC31A1 in a lysosome‐dependent manner (Figure S3D,E) and promoted plasma membrane translocation and RAB7A colocalization of ATP7A (Figure S3F,G; Figure 6D). These data confirm that PRMT5 lies upstream of the VPS34‐SLC31A1/ATP7A axis to control copper levels.

**FIGURE 6:**
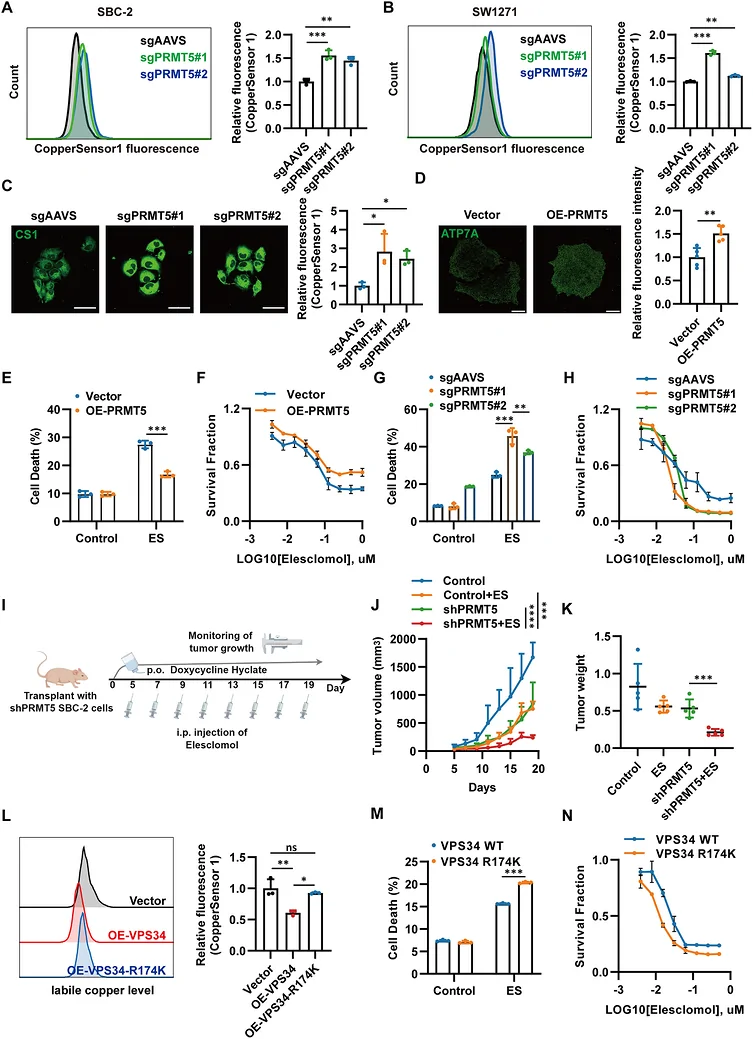
Methylation at Arg174 is essential for VPS34 stability and cellular resistance to cuproptosis. (A,B) Copper levels detection of CopperSensor 1 (CS1) in SBC‐2 and SW1271 cells with CRISPR/Cas9‐mediated deletion of PRMT5 following treatment with 1 µm CuCl2 in the medium. (C) Copper ions were analyzed with CS1 in the indicated SBC‐2 cells following treatment with 1 µm CuCl2 in the medium. *n* = 3 biologically independent experiments. Scale bars, 50 µm. (A–C) *p* values were calculated using one‐way ANOVA with Dunnett's multiple comparisons test (^*^p < 0.05, ^*^ ^*^p < 0.01, ^**^ ^*^p < 0.001). (D) Representative Total Internal Reflection Fluorescence (TIRF) microscopy images (left) and quantitative analysis of relative fluorescence intensity (right) of ATP7A in SBC‐2 cells expressing empty vector or OE‐PRMT5. Scale bars, 10 µm. (E) Levels of cell death in the indicated cells treated with 40 nm elesclomol in the presence of 1 µm CuCl2 in the medium for 72 h. (F) Viability of SBC‐2 cells overexpressing PRMT5 following treatment with elesclomol in the presence of 1 µm CuCl2 in the medium. (G) Levels of cell death among the indicated cells treated with 40 nm elesclomol in the presence of 1 µm CuCl2 in the medium for 72 h. (H) Viability of SBC‐2 cells with CRISPR/Cas9‐mediated deletion of PRMT5 following treatment with elesclomol in the presence of 1 µm CuCl2 in the medium. (E,G) The error bars represent the means ± SDs; n = 3 independent repeats. (E) *p* values were calculated using two‐tailed unpaired Student's t test (^**^ ^*^p < 0.001). (G) *p* values were calculated using one‐way ANOVA with Dunnett's multiple comparisons test (^*^ ^*^p < 0.01, ^**^ ^*^p < 0.001). (I) Illustration showing the workflow of the animal experiments. (J) Tumor volume curves for each group of nude mice. The error bars represent the means ± SDs; n = 5 independent repeats. P values were calculated using two‐way ANOVA (^*^ ^*^p < 0.01, ^**^ ^*^p < 0.001). (K) Average tumor weight in each group of nude mice. The error bars represent the means ± SDs; n = 5 independent repeats. *P* values were calculated using two‐tailed unpaired Student's t test (^**^ ^*^p < 0.001). (L) Copper levels in the indicated cells following treatment with 1 µm CuCl2 in the medium. *P* values were calculated using one‐way ANOVA with Tukey's multiple comparisons test (^*^p < 0.05, ^*^ ^*^p < 0.01, *ns*: *p* > 0.05). (M) Levels of cell death in the indicated cells treated with 40 nm elesclomol in the presence of 1 µm CuCl2 in the medium for 72 h. *P* values were calculated using two‐tailed unpaired Student's t test (^**^ ^*^p < 0.001). (N) Viability of the indicated cells following treatment with elesclomol in the presence of 1 µm CuCl2 in the medium.

We next evaluated the biological consequences of this regulation. The overexpression of PRMT5 significantly promoted resistance to ES‐Cu‐induced death (Figure 6E,F), whereas the depletion of PRMT5 sensitized cells to copper toxicity (Figure 6G,H). Further molecular analysis revealed that PRMT5 depletion significantly exacerbated DLAT oligomerization during cuproptosis, which could be reversed by FDX1 knockdown (Figure S3K,L). To validate these findings in vivo, we established a subcutaneous xenograft model using cells transduced with shPRMT5 or control shRNA. Compared with tumors in the control group, tumors in the PRMT5 knockdown group exhibited significantly lower growth rates and lower final tumor weights (Figure 6I–K; Figure S3H), indicating that PRMT5 is critical for tumor adaptation to copper stress in vivo.

Finally, to determine whether PRMT5 exerts its protective function specifically through the methylation‐dependent stabilization of VPS34, we performed a genetic rescue experiment. We reconstituted VPS34‐KO cells with either wild‐type (WT) VPS34 or a methylation‐deficient mutant (R174K). Western blotting confirmed that while WT‐VPS34 was stably expressed, the R174K mutant was unstable (data previously shown in Figure 5) and failed to accumulate to endogenous levels. Functionally, the re‐expression of WT‐VPS34 successfully restored the ability of the cells to pump out copper, reducing the intracellular copper concentration. In sharp contrast, the R174K mutation failed to reduce copper accumulation (Figure 6L; Figure S3I,J). Similarly, while WT‐VPS34 rescued the lethal phenotype and restored resistance to cuproptosis, cells expressing the R174K mutation remained highly sensitive to copper stress (Figure 6M,N). Taken together, these findings indicate that the PRMT5‐mediated dimethylation of VPS34 plays a critical role in maintaining intracellular copper homeostasis and inhibiting cuproptosis.

### 3.6 The PRMT5‐VPS34 Axis Contributes to Radioresistance

Given that ionizing radiation (RT) induces oxidative stress, which is intrinsically linked to metal ion homeostasis [6], we investigated the interplay between RT and intracellular copper. We observed that RT treatment caused a dose‐dependent increase in the intracellular copper concentration (Figure 7A). To determine the functional relevance of this copper surge, we modulated copper levels during RT. ES‐Cu supplementation significantly sensitized cells to RT, whereas chelation of copper using TTM protected cells from radiation‐induced death (Figure 7B,C), suggesting that excessive copper contributes to RT cytotoxicity. Strikingly, tumor cells appeared to mount an adaptive defense against this RT‐induced copper stress. Western blotting revealed that RT treatment triggered dose‐dependent upregulation of PRMT5 and VPS34 protein levels, accompanied by a robust increase in R174 dimethylation (Figure 7D). These data suggest that the PRMT5‐VPS34 axis is an inducible stress response pathway activated by radiotherapy.

**FIGURE 7:**
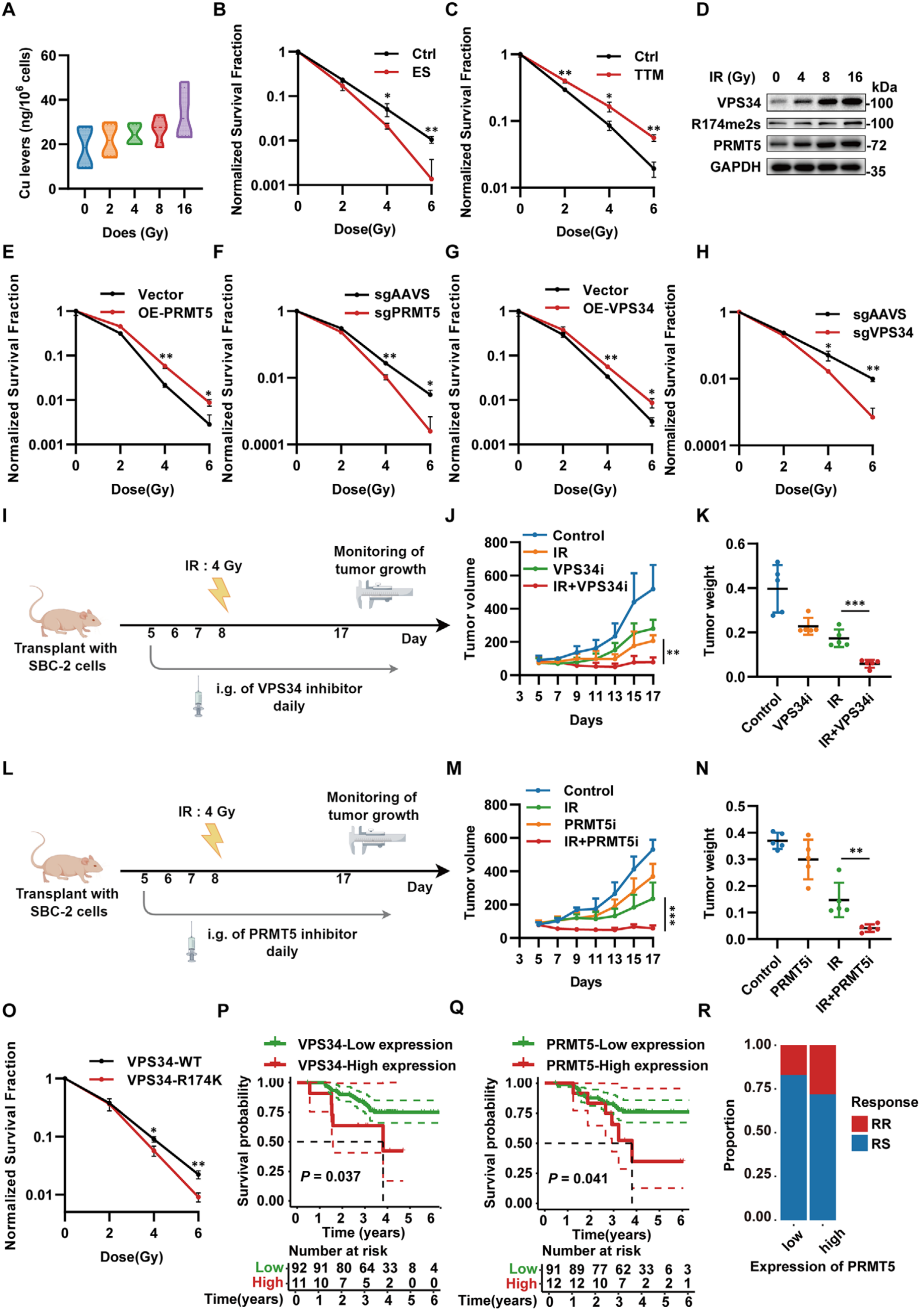
The PRMT5‐VPS34 axis contributes to radioresistance. (A) Copper levels were assessed via ICP‒MS in SBC‐2 cells following irradiation (n = 3). (B) Quantification of the clonogenic survival curve of SBC‐2 cells pretreated with or without elesclomol followed by exposure to IR at the indicated doses. (C) Quantification of the clonogenic survival curve of SBC‐2 cells pretreated with or without TTM followed by exposure to IR at the indicated doses. (D) Protein content in SBC‐2 cells following the indicated treatment. Data are representative of *n* = 3 biologically independent experiments. (E) The quantified clonogenic survival curve for SBC‐2 cells treated with irradiation at the indicated doses following PRMT5 overexpression. The error bars represent the means ± SDs; n = 3 biologically independent repeats. *P* values were calculated using multiple unpaired t tests (^*^ *p* < 0.05; ^**^, *p* < 0.01). (F) The quantified clonogenic survival curve for SBC‐2 cells treated with irradiation at the indicated doses following CRISPR/Cas9‐mediated deletion of PRMT5. (G) The quantified clonogenic survival curve for SBC‐2 cells treated with irradiation at the indicated doses following VPS34 overexpression. The error bars represent the means ± SDs; n = 3 biologically independent repeats. P values were calculated using multiple unpaired t tests (^*^, *p* < 0.05; ^**^, *p* < 0.01). (H) The quantified clonogenic survival curve for SBC‐2 cells treated with irradiation at the indicated doses following CRISPR/Cas9‐mediated deletion of VPS34. (I,L) Illustration showing the workflow of the animal experiments. (J,M) Tumor volume curves for each group of nude mice. The error bars represent the means ± SDs; n = 5 independent repeats. P values were calculated using two‐way ANOVA (^*^ ^*^p < 0.01, ^**^ ^*^p < 0.001). (K,N) Average tumor weight in each group of nude mice. The error bars represent the means ± SDs; n = 5 independent repeats. P values were calculated using two‐tailed unpaired Student's t test (^*^p < 0.05, ^**^ ^*^p < 0.001). (O) The quantified clonogenic survival curve for SBC‐2 cells treated with irradiation at the indicated doses following wild‐type (WT) VPS34 or R174K‐mutant VPS34 (VPS34‐R174K) overexpression. The error bars represent the means ± SDs; *n* = 3 biologically independent repeats. P values were calculated using multiple unpaired t tests (^*^p < 0.05). (P,Q) Kaplan‒Meier survival analysis of OS for lung cancer patients in GSE32649877 with low and high protein expression of VPS34 and PRMT5. The R package “survminer” was used to determine the best cutoff gene expression for survival analysis. (R) Correlations between PRMT5 expression and treatment response to radiotherapy in pancancer patients in the TCGA. Patients with a complete response or partial response were defined as radiosensitization (RS), and those with progressive disease or stable disease were defined as radioresistance (RR). The R package “survminer” was used to determine the best cutoff gene expression for this analysis. All the original blots can be found in Supporting File S2.

We next examined whether this pathway contributes to radioresistance using clonogenic survival assays. Genetic modulation confirmed the protective role of this axis: overexpression of VPS34 or PRMT5 significantly reduced radiosensitivity (increased survival), whereas their depletion markedly sensitized cells to RT (Figure 7E–H). To elucidate the mechanism underlying this resistance, we performed a rescue experiment in the context of radiation. Consistent with our copper homeostasis findings, reconstitution with WT‐VPS34 resulted in significant radioresistance, whereas the methylation‐deficient R174K mutant failed to protect cells from RT‐induced death (Figure 7O). These findings indicate that the PRMT5‐mediated stabilization of VPS34 is essential for the development of resistance to radiotherapy.

To evaluate the therapeutic potential of targeting this axis, we employed pharmacological inhibitors in a subcutaneous xenograft model. Combination therapy yielded potent synergistic effects: compared with RT alone, treatment with a specific VPS34 inhibitor significantly sensitized tumors to RT, resulting in suppressed tumor growth and reduced tumor burden (Figure 7I–K; Figure S4A). Similarly, the administration of a PRMT5 inhibitor markedly enhanced the efficacy of radiotherapy (Figure 7L–N; Figure S4B). These in vivo data strongly suggest that pharmacological blockade of the PRMT5‐VPS34 axis can overcome radioresistance.

Finally, we assessed the clinical significance of our findings using public patient datasets. High protein expression levels of PRMT5 and VPS34 were significantly associated with poor overall survival in cancer patients (Figure 7P,Q). Furthermore, analysis of data from the TCGA database revealed that patients with high PRMT5 mRNA expression exhibited a significantly poorer response to radiotherapy (Figure 7R). Collectively, these clinical and experimental data support a model in which the PRMT5‐VPS34 signaling axis functions as a critical adaptive mechanism that limits therapeutic efficacy, highlighting it as a promising target for radiosensitization.

## 4 Discussion

While cuproptosis represents a potent therapeutic vulnerability, its clinical exploitation is currently hampered by an incomplete understanding of how malignant cells navigate copper‐induced cytotoxicity [3]. Identifying the molecular “brakes” that suppress this pathway is essential for developing rational sensitizing strategies. Through a genome‐wide CRISPRa screen, we identified the PRMT5‐VPS34 axis as a pivotal regulatory network that shields cancer cells from copper‐induced proteotoxicity. Our findings establish that the PRMT5‐mediated stabilization of VPS34 constitutes a previously unrecognized mechanism of cuproptosis evasion and critically serves as a prerequisite for adaptive resistance to radiotherapy (Figure S4C).

It is well known that the execution of cuproptosis is driven by the oligomerization of lipoylated proteins (such as DLAT) and is regulated by key metabolic enzymes such as FDX1 [16, 17]. However, as this form of cell death is strictly dependent on copper availability, the upstream machinery that dictates copper influx and efflux during stress remains a critical determinant of cell fate [2]. Recent studies have shown that radiotherapy triggers a lethal surge in intracellular copper levels by upregulating the expression of the importer SLC31A1 [6]. Our study revealed that the PRMT5‐VPS34 axis functions as an upstream “gatekeeper” of intracellular copper levels. By producing PI3P, VPS34 orchestrates a bimodal trafficking strategy: it selectively directs the importer SLC31A1 for lysosomal degradation while simultaneously facilitating the recycling of the exporter ATP7A to the plasma membrane via RAB7A‐positive endosomes. This dual action ensures rapid clearance of cytosolic copper, effectively limiting potentially lethal copper accumulation induced by therapeutic stress.

Our identification of the PRMT5‐VPS34 axis as a critical suppressor of cuproptosis reveals a distinct therapeutic vulnerability for overcoming radioresistance. While small‐molecule inhibitors of VPS34 (such as SAR405) have been developed [18], their clinical application remains largely restricted to preclinical autophagy studies, and no VPS34‐targeted therapies are currently approved for cancer treatment. In contrast, PRMT5 inhibitors (e.g., GSK3326595 and JNJ‐64619178) are rapidly emerging as a potent class of therapeutics, with several candidates currently advancing through phase I/II clinical trials [19, 20, 21]. However, existing trials have focused predominantly on PRMT5 inhibitors as monotherapies or in combination with chemotherapy; to date, the therapeutic potential of combining PRMT5 inhibition with radiotherapy remains largely unexplored [11]. Our discovery provides the missing mechanistic link to bridge this gap. We established that PRMT5 acts as an essential upstream stabilizer of VPS34 by mediating its symmetric dimethylation at Arg174. This specific modification recruits the deubiquitinase USP10, thereby shielding VPS34 from K48‐linked polyubiquitination and proteasomal turnover. Given that this axis functions as a “homeostatic reserve” in aggressive cancers, we propose that PRMT5 inhibitors should be strategically repositioned as radiosensitizers. By pharmacologically dismantling the PRMT5‐VPS34 axis, we can convert the sublethal stress of radiotherapy into lethal cuproptosis, providing a robust rationale for a novel combinatorial regimen.

Despite the significant findings, we acknowledge several limitations in our current study. First, our mechanistic investigations primarily rely on specific lung cancer models, particularly the SBC‐2 cell line. While we elucidated the VPS34‐dependent regulation of SLC31A1 and ATP7A in this context, the broader applicability of this mechanism to other tissues with distinct copper handling machinery—such as the liver, where ATP7B is predominant—remains unclear and requires further validation. Second, although we demonstrated that copper and radiation stress robustly upregulate PRMT5, the specific upstream sensing machinery remains to be fully characterized. Previous studies have reported that reactive oxygen species (ROS) can drive PRMT5 upregulation in other contexts, such as through Nox4 induction, and ionizing radiation is a well‐known ROS‐generating stimulus [22, 23, 24]. However, our current study lacks direct experimental evidence to confirm whether a specific ROS‐PRMT5 axis or distinct transcription factors mediate this process in our specific lung cancer models. Therefore, delineating these upstream mechanisms represents another limitation of our current work and an important direction for future research.

In summary, we demonstrate that PRMT5 enhances VPS34 stability via methylation‐dependent recruitment of USP10, which in turn coordinates vesicle trafficking to prevent copper overload. This PRMT5‐mediated suppression of cuproptosis is an underappreciated feature of radioresistant cancers. Our findings underscore that cuproptosis resistance plays a pivotal role in the failure of standard therapies. Deciphering the diverse mechanisms by which malignant tumors resist metal toxicity and developing targeted strategies to breach these defenses will be key to successfully implementing cuproptosis‐based therapies in the clinical management of cancer.

## Author Contributions

The order of the co‐first authors was determined on the basis of the significance, time, and effort each author invested in the project. **Yuan‐Yuan Chen**, **Xuan Li**, **Ming Chen**, and **Qi‐Wen Li** supervised and reviewed the study. **Yuan‐Yuan Chen**, **Xuan Li**, and **Wei Chen** designed the study. **Wei Chen**, **Ming‐Ying Xiao**, **Lan‐Lan Guo**, and **Hao‐Yue Hu** performed most of the experiments with the assistance form **Sui‐Xian Zhang**, **Bai‐Qiang Dong**, **Chen‐Fei Wu**, **Xia‐Hang Huang**, **Li Li**, **Run‐Zhe Chen**, **Yuan‐Yang Huang**, **Ying Wang**, **Wei Chen**, **Ming‐Ying Xiao**, and **Lan‐Lan Guo** contributed to the acquisition, analysis, or interpretation of the data. **Wei Chen**, **Ming‐Ying Xiao**, **Lan‐Lan Guo**, and **Hao‐Yue Hu** contributed to manuscript drafting. All authors were involved in the critical revision of the manuscript for important content and approved the final manuscript.

## Funding

This study was supported by the Noncommunicable Chronic DiseasesNational Science and Technology Major Project (Grant No. 2023ZD0502100 [to M.C.]), National Key R&D Program of China (Grant No. 2023YFC2413900 [to M.C.]), Natural Science Foundation of China (Grant No. 82272744 [to M.C.], 82372700 [to X.L.], 81802789 [to X.L.], 82202827 [to R.‐Z.C.], 82303577 [to B.‐Q.D], 82303693 [to C.‐F.W], 82103437 [to Y.W.]), Cancer Innovative Research Program of Sun Yat‐sen University Cancer Center (Grant No. PT13030302 [to M.C.]), Natural Science Foundation of Guangdong Province (Grant No. 2022A1515010814 [to M.C.]), the Fundamental Research Funds for the Central Universities, Sun Yatsen University (Grant No. 2022008 [to Y.‐Y.C.]), Young Talents Program of Sun Yat‐sen University Cancer Center (Grant No. YTP‐SYSUCC0064 [to X.L.]), Guangzhou Science and Technology Plan (Grant No. 2023A04J1771 [to X.L.]), Guangzhou Basic and Applied Basic Research Foundation (Grant No. 202201010918 [to Y.W.]), and China Postdoctoral Science Foundation (Grant No. 2018M640866 [to X.L.]). The funders had no role in the study design, data collection and analysis, decision to publish, or preparation of the manuscript.

## Ethics Statement

All Animal Care and Experimental Procedures Were Approved by the Institutional Animal Care and Use Committee (IACUC) of Sun Yat‐Sen University Cancer Center (Approval No. L025501202308014).

## Conflicts of Interest

The authors declare no conflicts of interest.

## Supporting information




**Supporting File 1**: advs76350‐sup‐0001‐SuppMat.docx.

**Supporting File 2**: advs76350‐sup‐0002‐TableS1.csv.

**Supporting File 3**: advs76350‐sup‐0003‐DataFile.pdf.

## Data Availability

The AUC values for the elesclomol and proteomic data of the pancancer cell lines were obtained from the Cancer Cell Line Encyclopedia (CCLE; https://depmap.org/portal/). Data for evaluating the prognostic value of VPS34 and PRMT5 protein expression were acquired from the Gene Expression Omnibus (GSE32649877), while data concerning the relationship between the expression of PRMT5 and radiotherapy sensitivity were acquired from The Cancer Genome Atlas (TCGA, https://portal.gdc.cancer.gov/). All other data supporting the findings of this study are available from the corresponding author upon reasonable request.
