# Supplementary material for: PRMT5‐Dependent Stabilization of VPS34 Orchestrates Copper Trafficking to Shield Cancer Cells from Cuproptosis and Radiotherapy
Source: Adv Sci (Weinh). 2026 Jul 8;13(53):e76350. doi: 10.1002/advs.76350 (PMC13345692; doi:10.1002/advs.76350)
Supplement: Supplementary file 2 [file ADVS-13-e76350-s003.docx]

**Supplementary Figure 1 Autophagy protects cells from cuproptosis.** (A) Protein content in SBC-2 cells following treatment with 100 nM Baf A1 (6 h) and 40 nM elesclomol (24 h). (B) Confocal immunofluorescence imaging (LC3, green; p62, red; and DAPI, blue). (C) Foci were segmented and quantified in each condition. The error bars represent the means ± SDs; n = 5 independent repeats. (D) Copper levels were assessed via ICP‒MS in SBC-2 cells following the indicated treatments (n = 3). (E) Viability of SBC-2 cells treated with DMSO, CQ or Baf A1. (F) Levels of cell death among SBC-2 cells treated with DMSO, 10 µM CQ or 100 nM Baf A1 for 24 h. The error bars represent the means ± SDs; n = 3 independent replicates. *P* values were calculated using one-way ANOVA with Dunnett’s multiple comparisons test (*** P* < 0.01, **** P* < 0.001). (G) Illustration showing the workflow of the animal experiments. (H) Tumor volume curves for each group of nude mice. The error bars represent the means ± SDs; n = 5 independent repeats. P values were calculated using two-way ANOVA (*** P* < 0.01, **** P* < 0.001). (I) Average tumor weight in each group of nude mice. The error bars represent the means ± SDs; n = 5 independent repeats. P values were calculated using two-tailed unpaired Student’s t test (** P* < 0.05, **** P* < 0.001). (J) Image of tumors excised at the end of the experiment. (K, L) The DLAT oligomer levels in the indicated SBC-2 cells with or without the treatment of 40 nM elesclomol (1 µM CuCl2). All western blot data are representative of *n* = 3 biologically independent experiments. All the original blots can be found in Supporting File 2.

**Supplementary Figure 2 VPS34 maintains intracellular copper homeostasis.** (A-B) Copper levels in SBC-2 or SW1271 cells expressing empty vector or with VPS34 overexpression following treatment with 1 µM CuCl2 in the medium. *n* = 3 biologically independent experiments. (C) Copper ions were analyzed with Coppersensor-1 (CS1) in the indicated SBC-2 cells following treatment with 1 µM CuCl2 in the medium. Scale bars, 50 µm. *n* = 3 biologically independent experiments.

(D, E) Quantitative colocalization analysis using Manders’ Colocalization Coefficients (MCC). Specifically, M1 represents the fraction of red signal (LAMP1, LC3, etc.) overlapping with green (ATP7A), while M2 represents the fraction of green overlapping with red. *n* = 3 biologically independent experiments. (A-E) Data are presented as mean ± SD. ** P* < 0.05, ** *P* < 0.01, *ns*: *P* > 0.05, as determined by unpaired two-tailed Student's t-test. (F) Left: representative confocal immunofluorescence images showing the subcellular distribution of ATP7A (green) and LAMP1 (red) in control and VPS34-overexpressing cells. Right: colocalization analysis of ATP7A with the lysosome marker LAMP1 using ImageJ. (G) Left: Representative confocal immunofluorescence images showing the subcellular distribution of ATP7A (green) and LC3 (red) in control and VPS34-overexpressing cells. Right: colocalization analysis of ATP7A with LC3 using ImageJ. All the original blots can be found in Supporting File 2.

**Supplementary Figure 3 Methylation at Arg174 is essential for VPS34 stability and cellular resistance to cuproptosis.** (A-B) Copper levels in SBC-2 or SW1271 cells expressing empty vector or with PRMT5 overexpression following treatment with 1 µM CuCl2 in the medium. *n* = 3 biologically independent experiments. (C) Copper ions were analyzed with CS1 in the indicated SBC-2 cells expressing empty vector or with PRMT5 overexpression following treatment with 1 µM CuCl2 in the medium. Scale bars, 50 μm. *n* = 3 biologically independent experiments. (A-C) Data are presented as mean ± SD. *** P* < 0.01 , as determined by unpaired two-tailed Student's t-test. (D) Western blot showing the expression of copper transporters in the indicated cells with empty vector expression or PRMT5 overexpression. (E) Western blot showing the expression of copper transporters in the indicated cells expressing empty vector or with PRMT5 overexpression following treatment with 500 nM Baf A1 for 12 h. (F) Left: Representative confocal immunofluorescence images showing the subcellular distribution of ATP7A (green) and RAB7A (red) in control and PRMT5-overexpressing cells. Right: Colocalization analysis of ATP7A with the endosome marker RAB7A using ImageJ. (G) Quantitative colocalization analysis using Manders’ Colocalization Coefficients (MCC). Specifically, M1 represents the fraction of red signal (RAB7A) overlapping with green (ATP7A), while M2 represents the fraction of green overlapping with red. *n* = 3 biologically independent experiments. Data are presented as mean ± SD. ** P* < 0.05, *ns*: *P* > 0.05, as determined by unpaired two-tailed Student's t-test. (H) Image of tumors excised at the end of the experiment. (I) Western blot showing the expression of VPS34 in the indicated cells overexpressing wild-type VPS34 (WT) or R174K VPS34 (R174K). (J) Copper ions were analyzed with CS1 in the indicated SBC-2 cells following treatment with 1 µM CuCl2 in the medium. Scale bars, 50 μm. *n* = 3 biologically independent experiments. Data are presented as mean ± SD. *P* values were calculated using one-way ANOVA with Tukey’s multiple comparisons test. (K, L) The DLAT oligomer levels in the indicated SBC-2 cells with or without the treatment of 40 nM elesclomol (1 µM CuCl2). All western blot data are representative of *n* = 3 biologically independent experiments. All the original blots can be found in Supporting File 2.

**Supplementary Figure 4 The PRMT5-VPS34 axis contributes to radioresistance.** (A, B) Left: Images of tumors excised at the end of the experiment. Right: Average body weight in each group of nude mice. The error bars represent the means ± SDs; n = 5 independent repeats. *P* values were calculated using two-way ANOVA (*ns*: *P* > 0.05). (C) Working model depicting the roles and mechanisms of cuproptosis in the IR response and radioresistance. See the Discussion for a detailed description. All the original blots can be found in Supporting File 2.
