# Supplementary material for: PRMT5‐Dependent Stabilization of VPS34 Orchestrates Copper Trafficking to Shield Cancer Cells from Cuproptosis and Radiotherapy
Source: Adv Sci (Weinh). 2026 Jul 8:e76350. Online ahead of print. doi: 10.1002/advs.76350 (PMC13345692; doi:10.1002/advs.76350)

Fig 1G

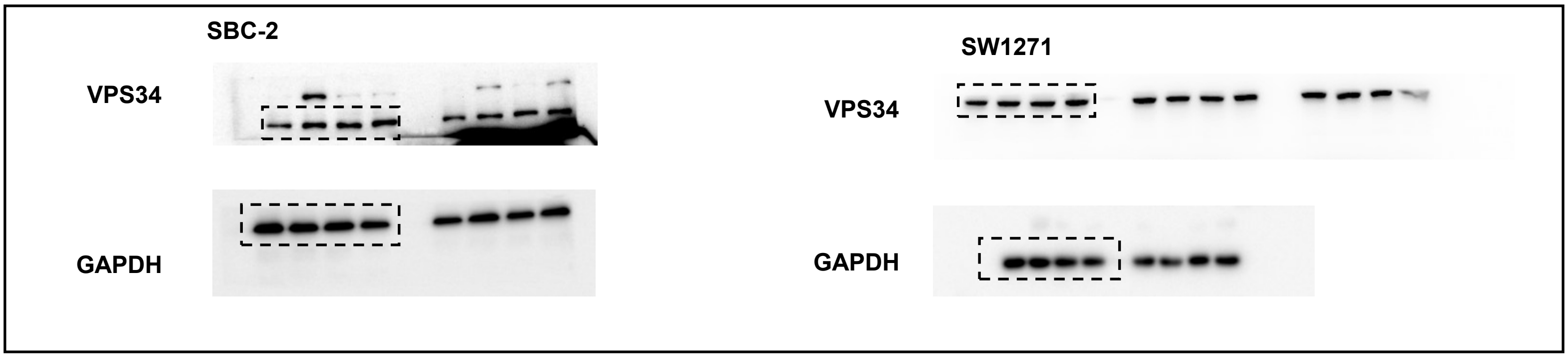

Fig 2D

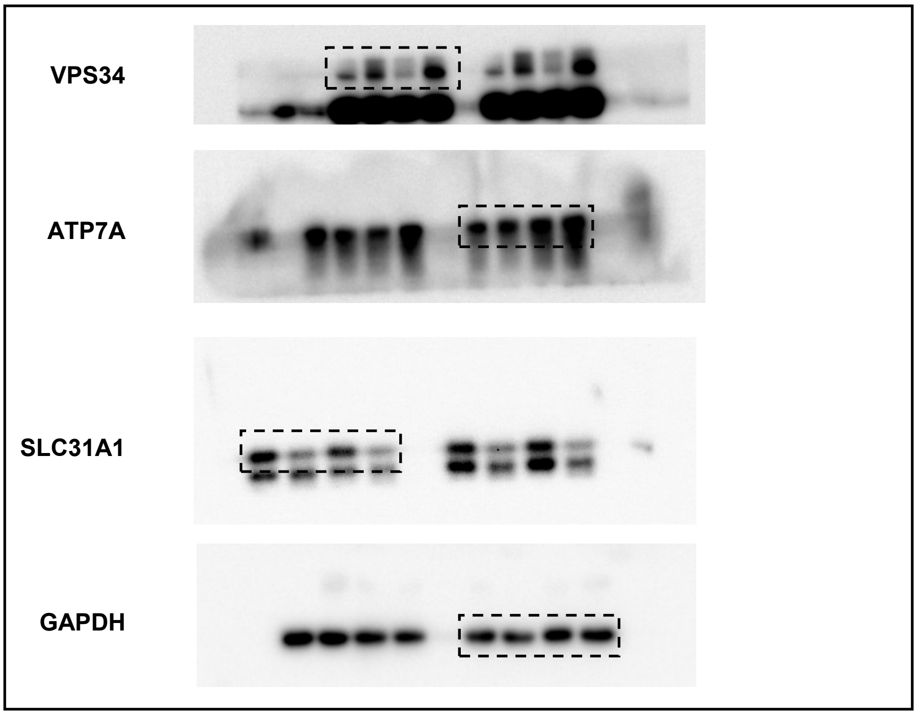

Fig 2E

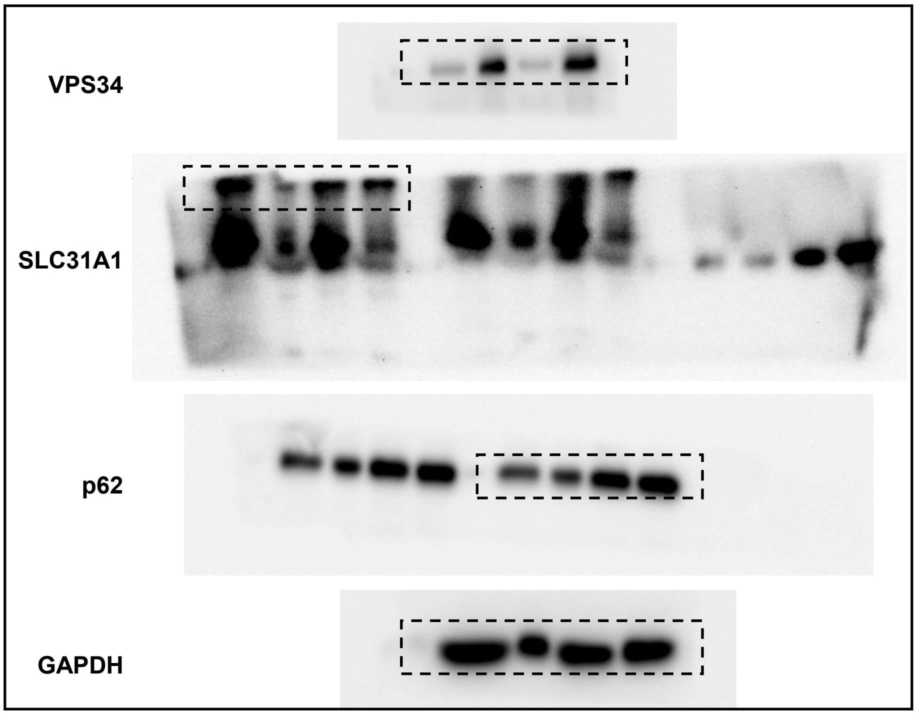

Fig 3C

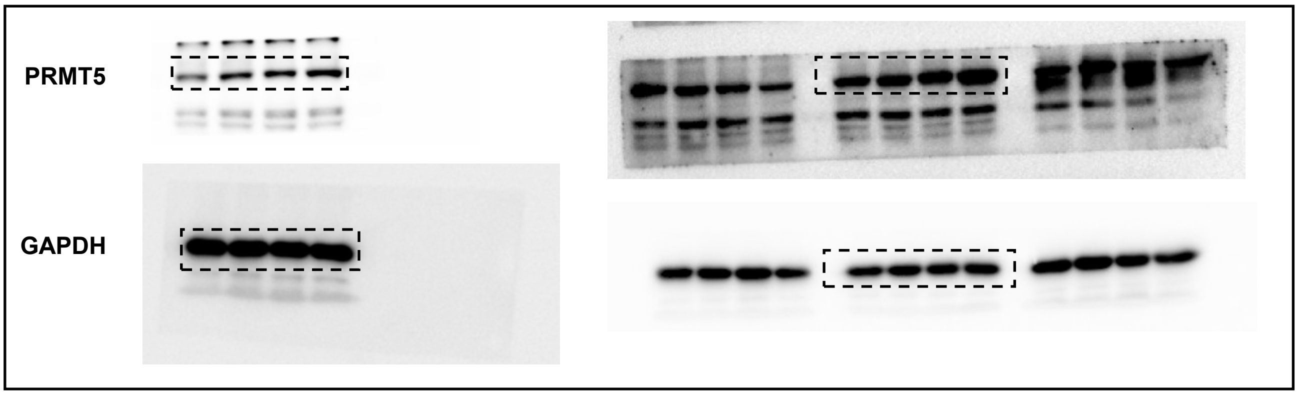

Fig 3D

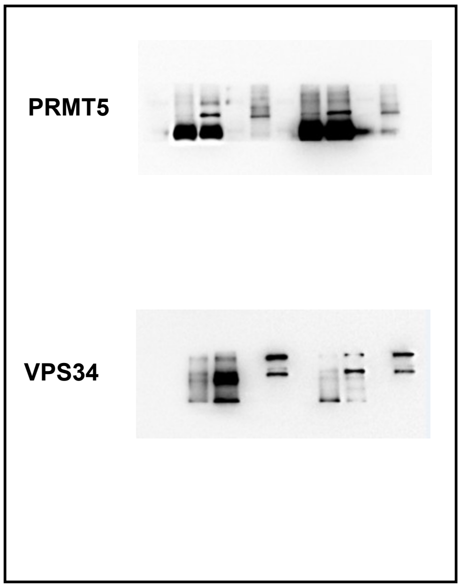

Fig 3E

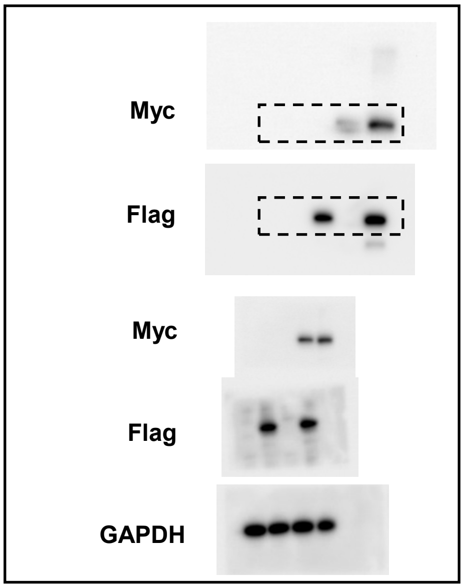

Fig 3F

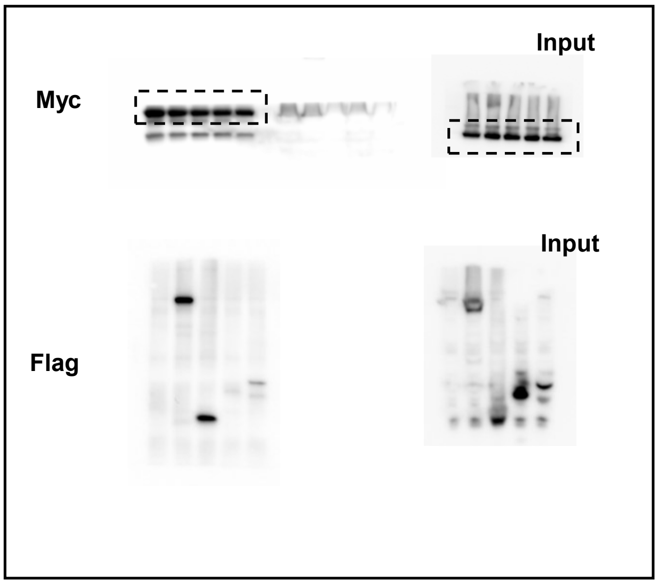

Fig 3G

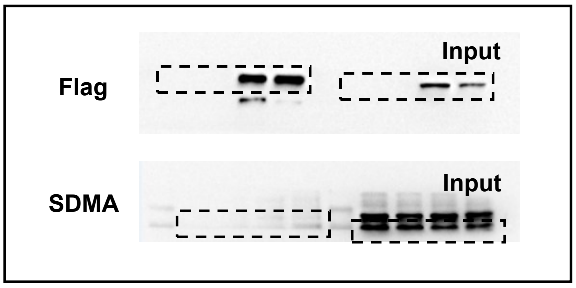

Fig 3H

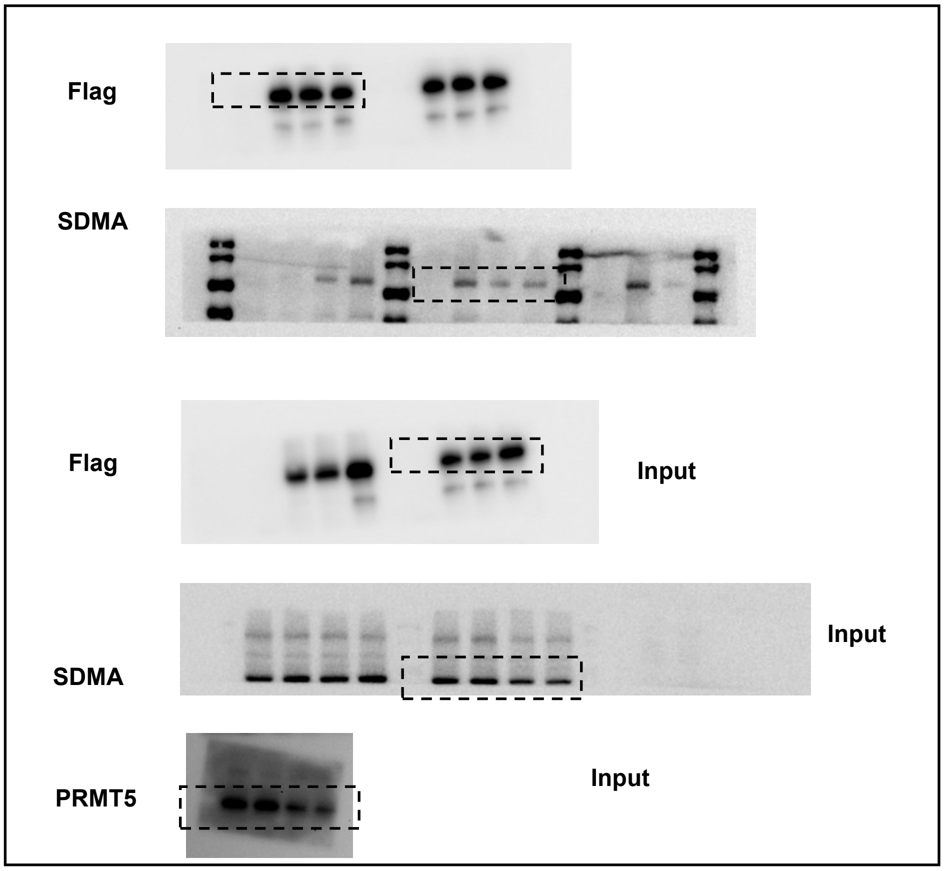

Fig 3K

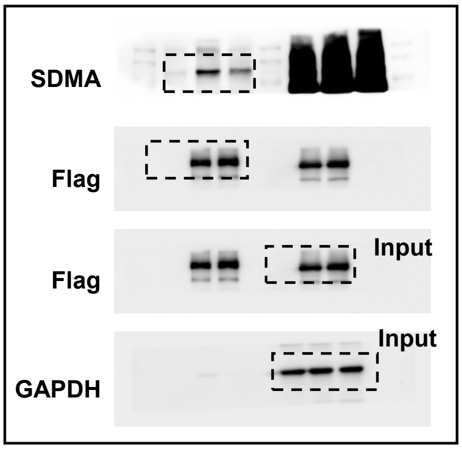

Fig 3L

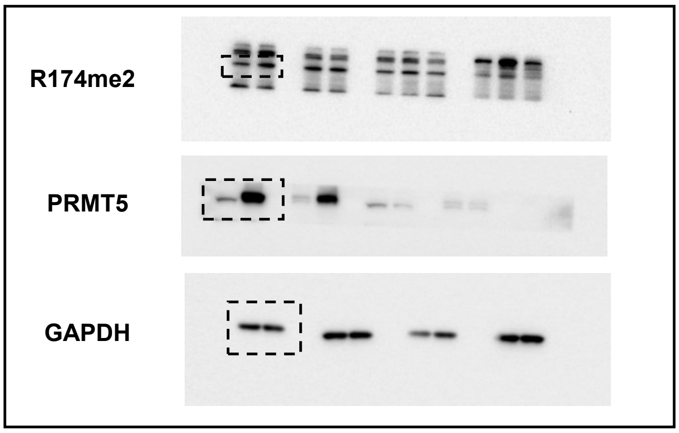

Fig 3M

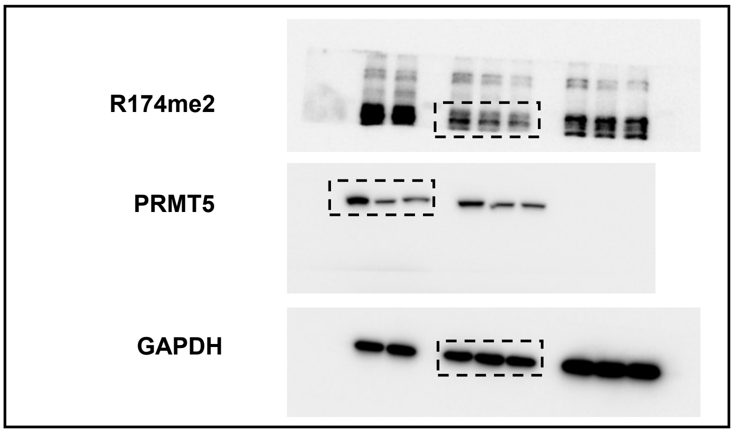

Fig 4B

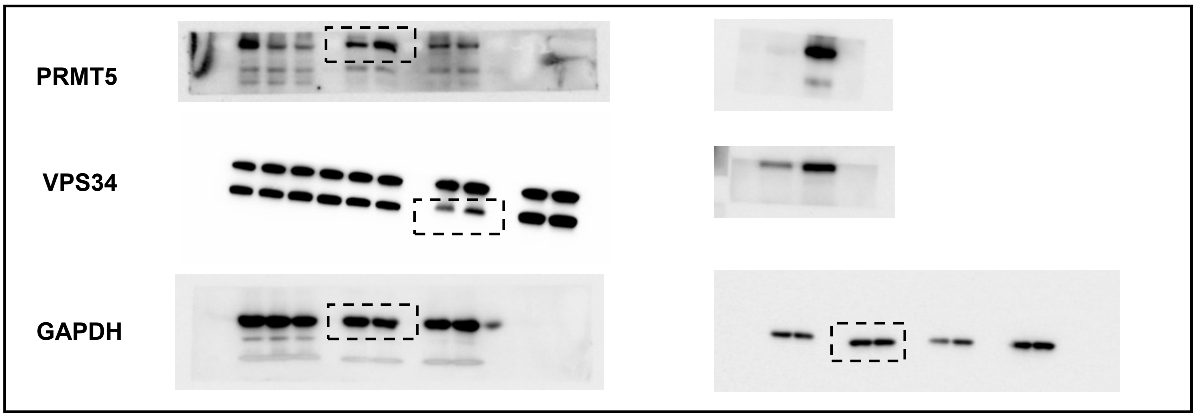

Fig 4D

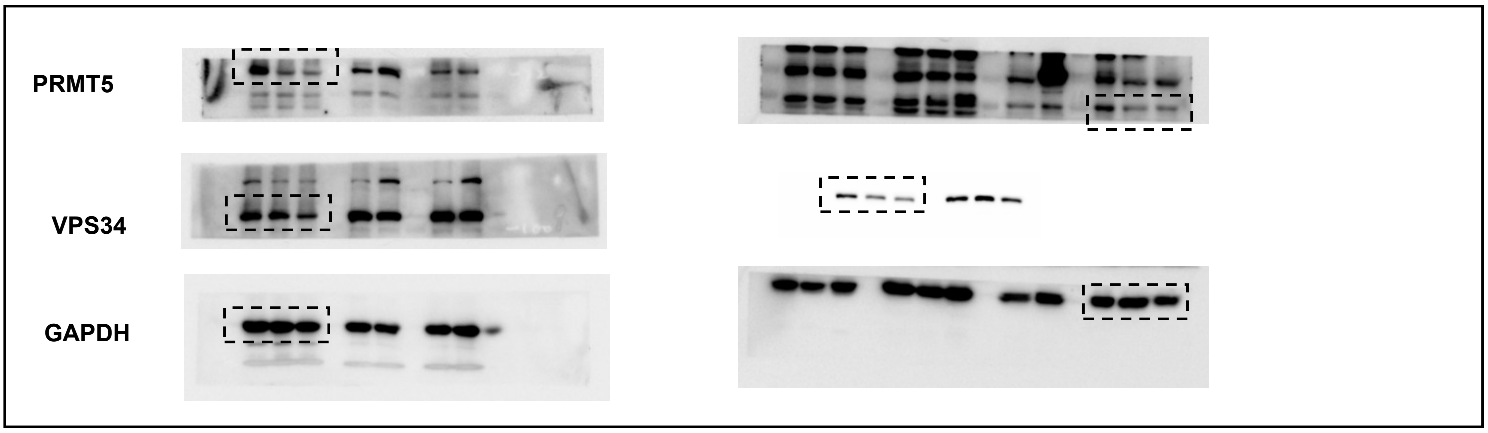

Fig 4E

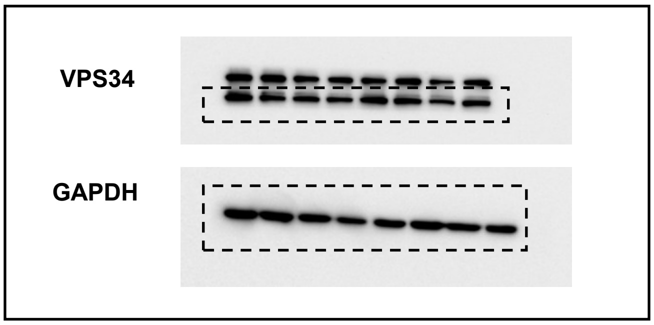

Fig 4F

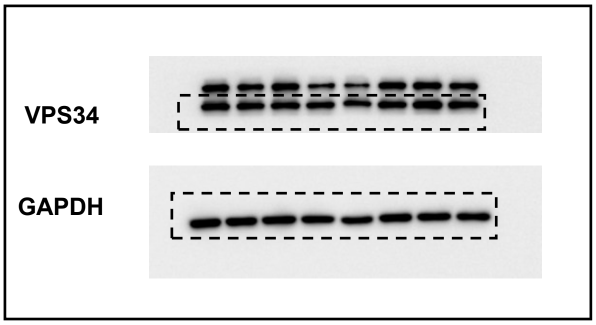

Fig 4G

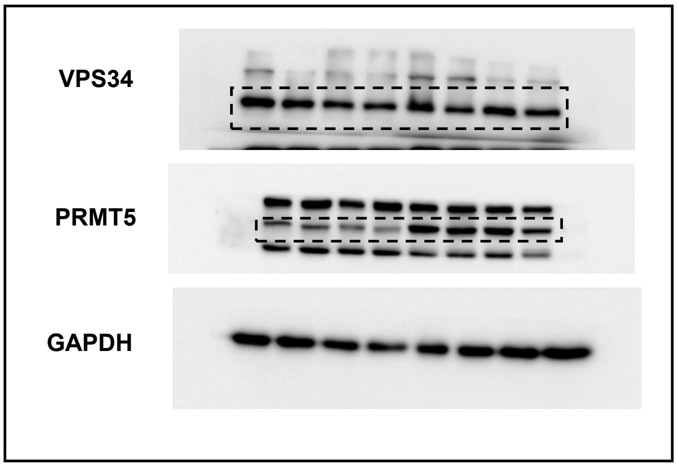

Fig 4H

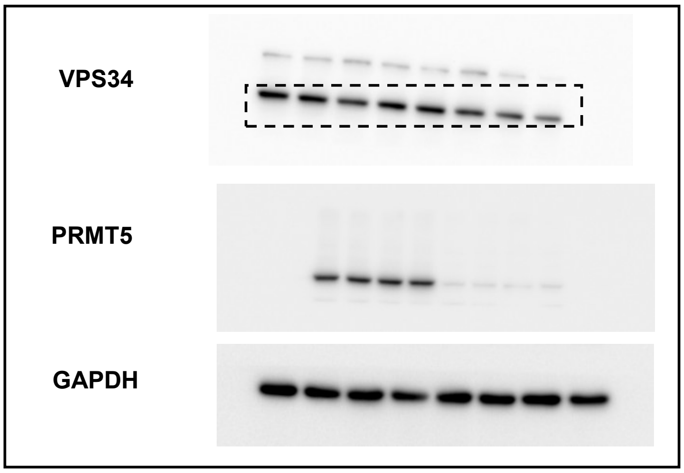

Fig 4I

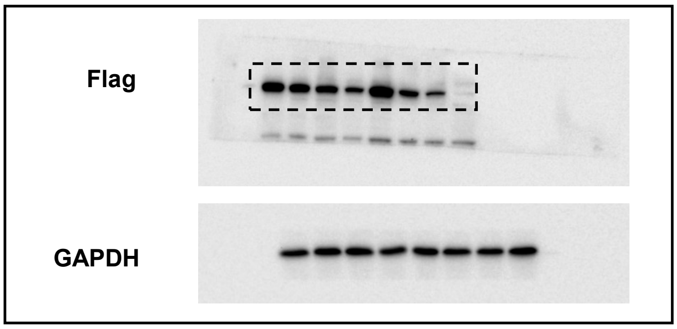

Fig 5A

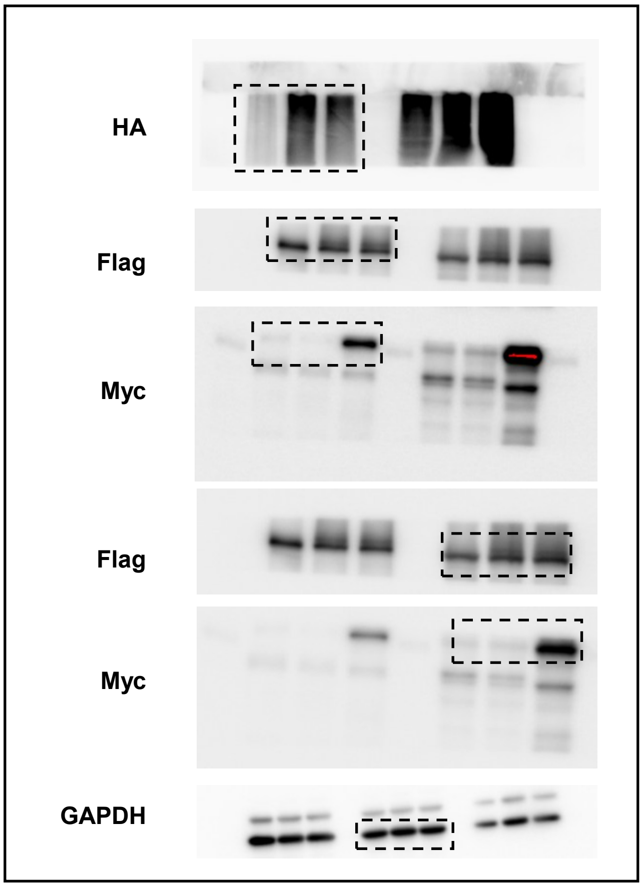

Fig 5B

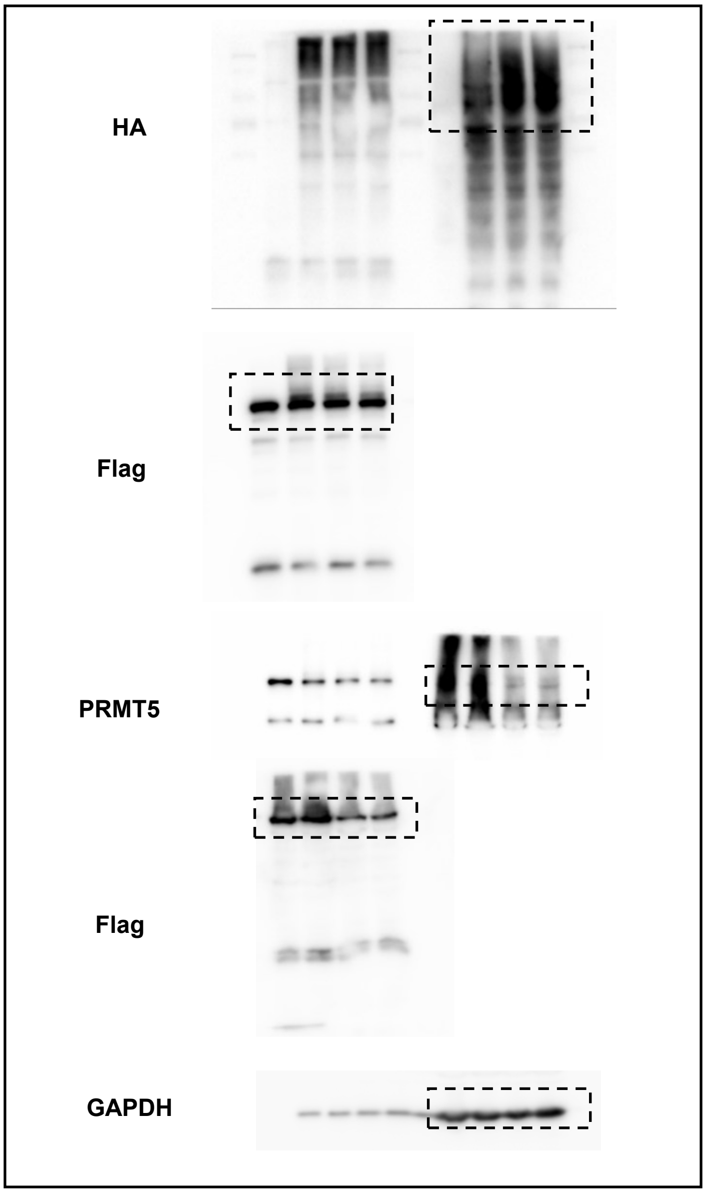

Fig 5C

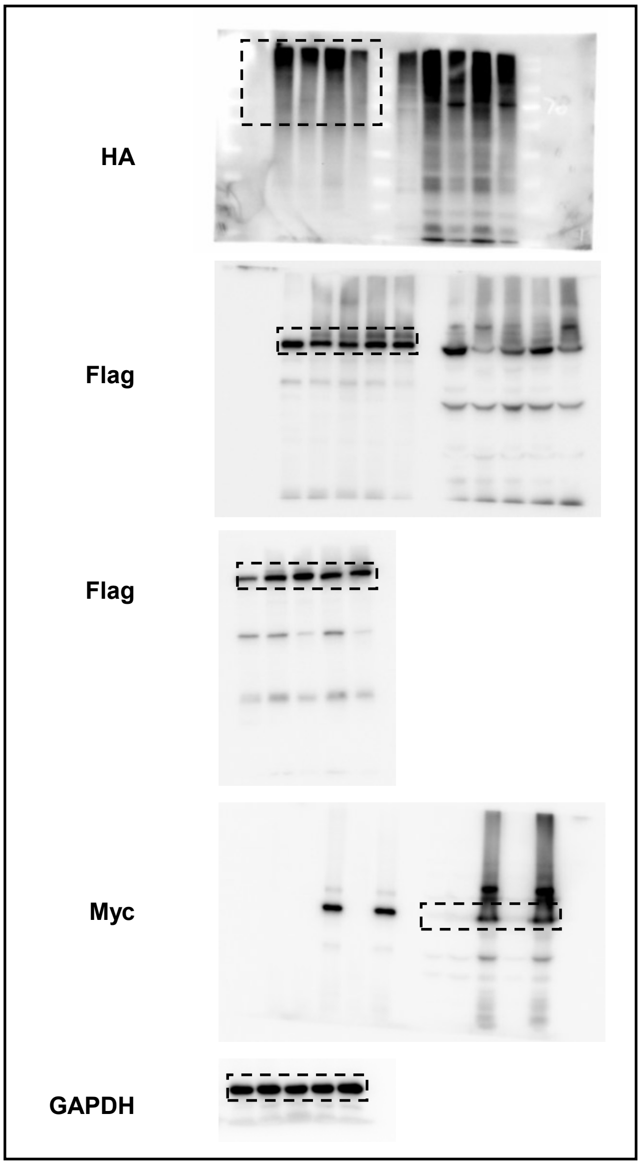

Fig 5D

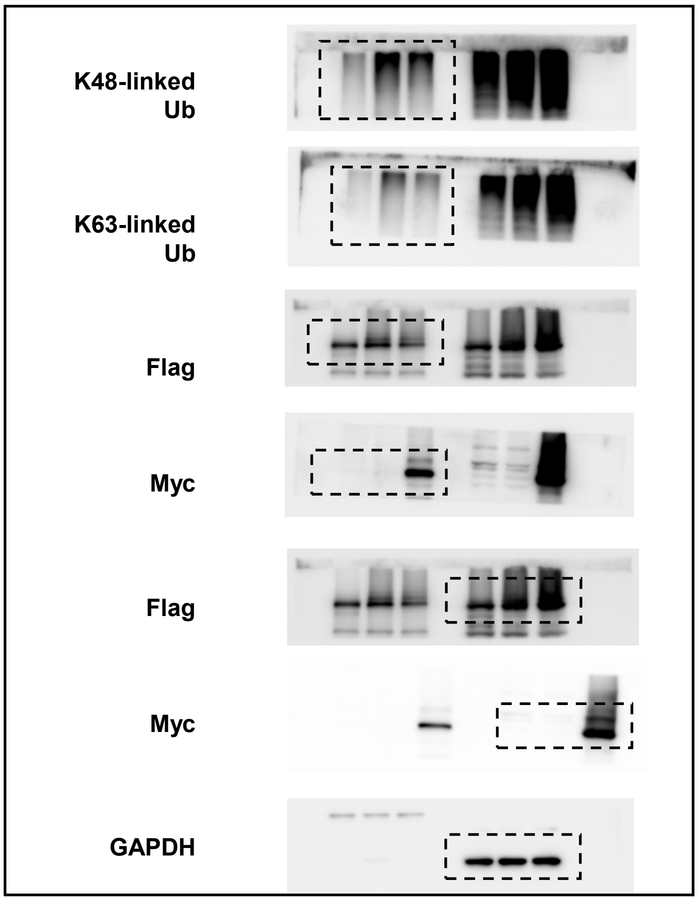

Fig 5E

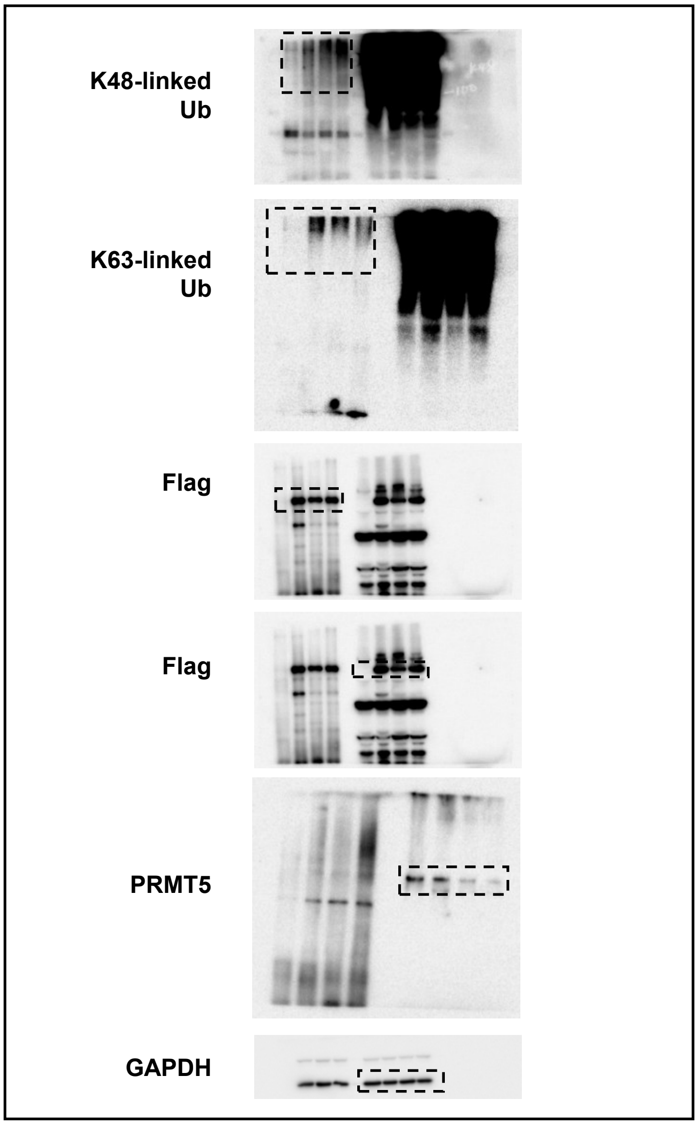

Fig 5F

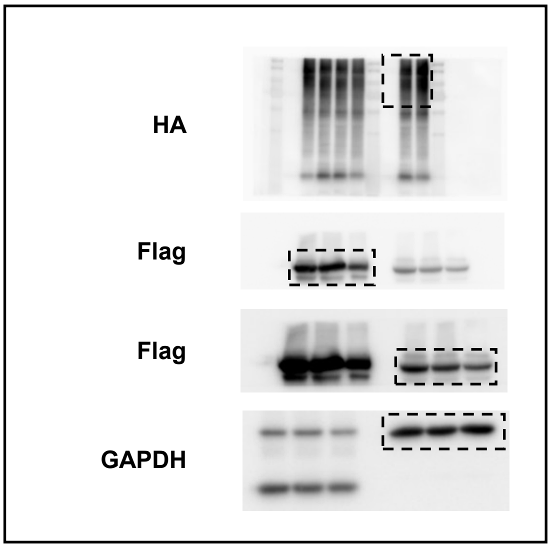

Fig 5H

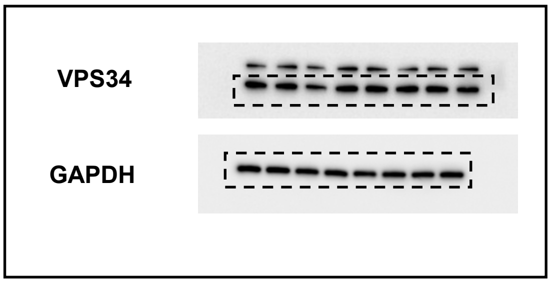

Fig 5I

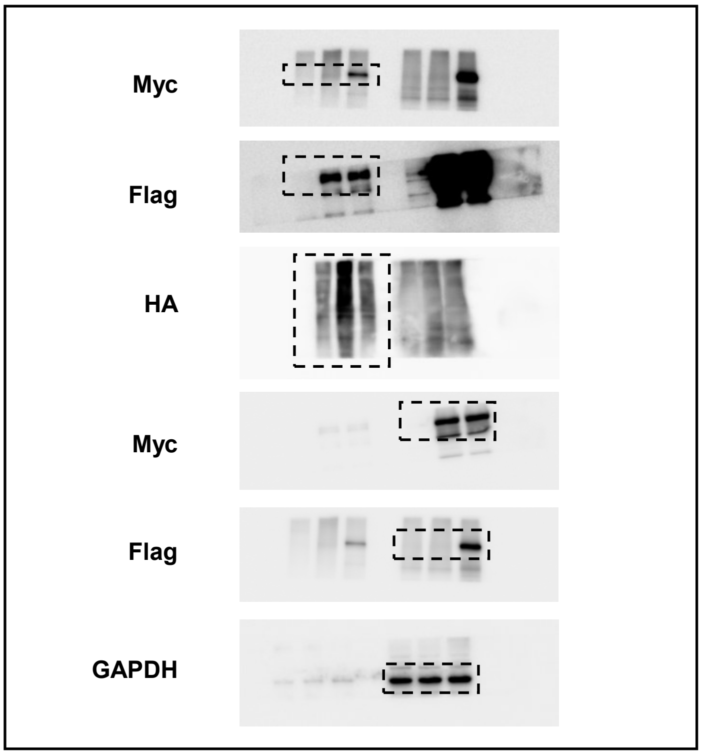

Fig 5J

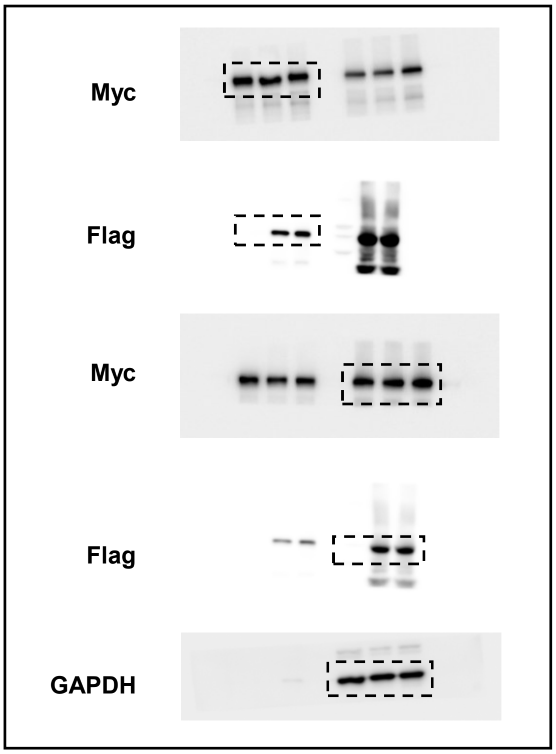

Fig 7D

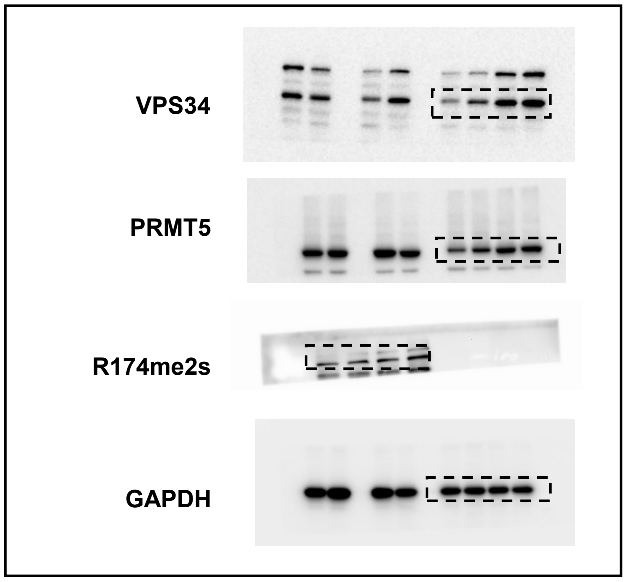

SFig 1A

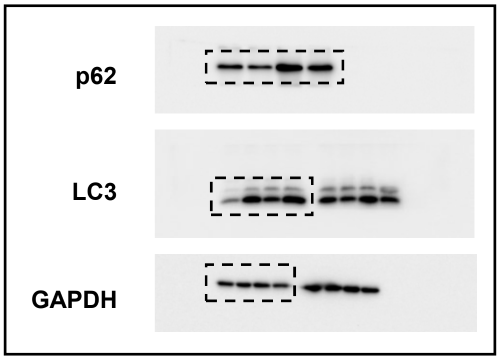

SFig 1K

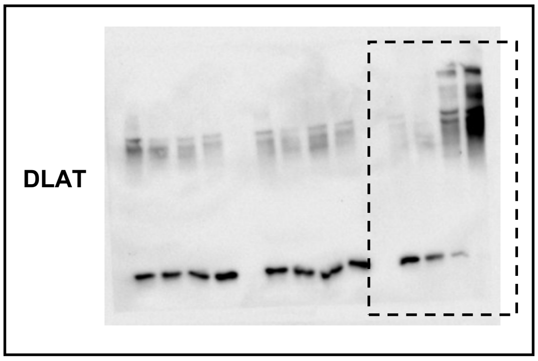

SFig 1L

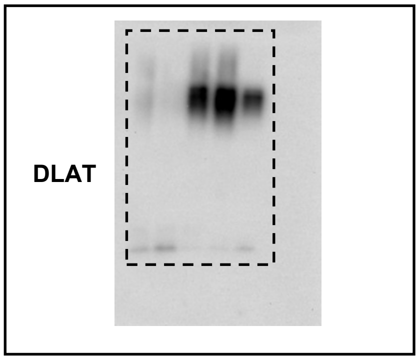

SFig 3D

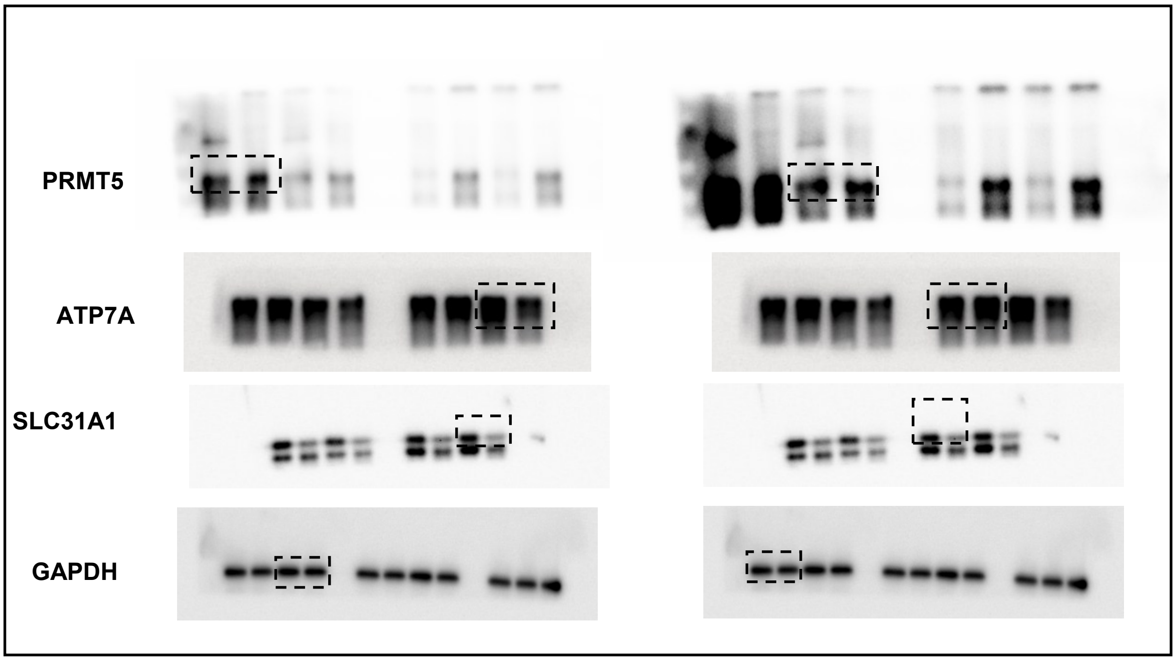

SFig 3I

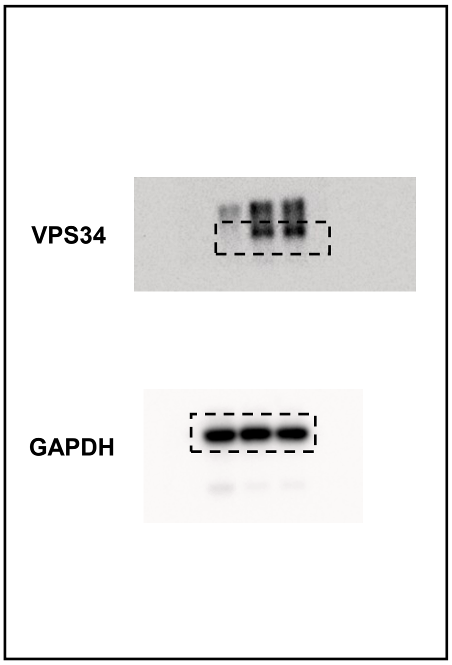

SFig 3E

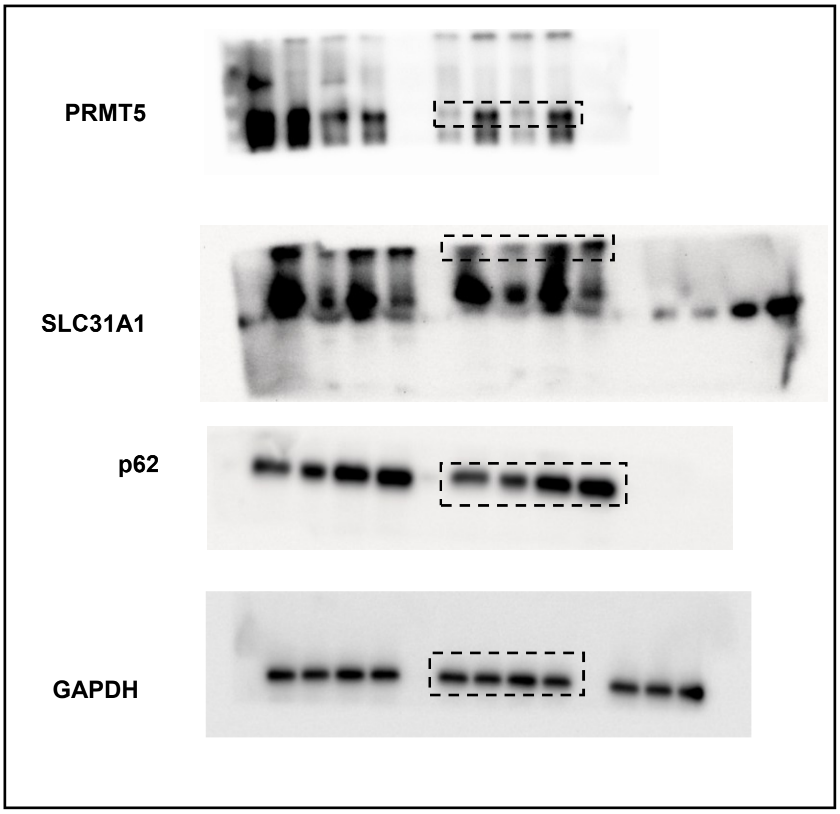

SFig 3K

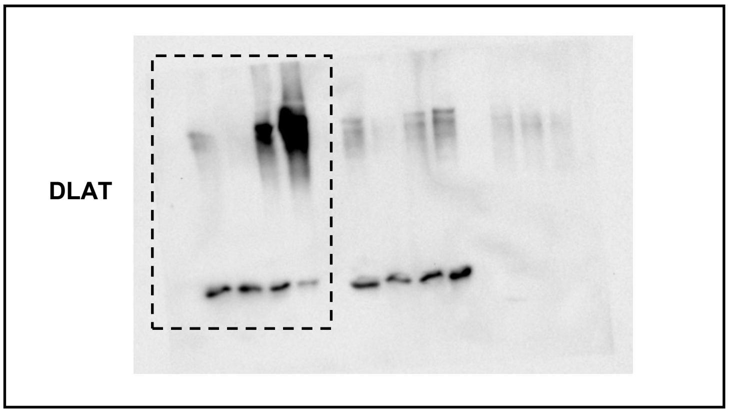

SFig 3L

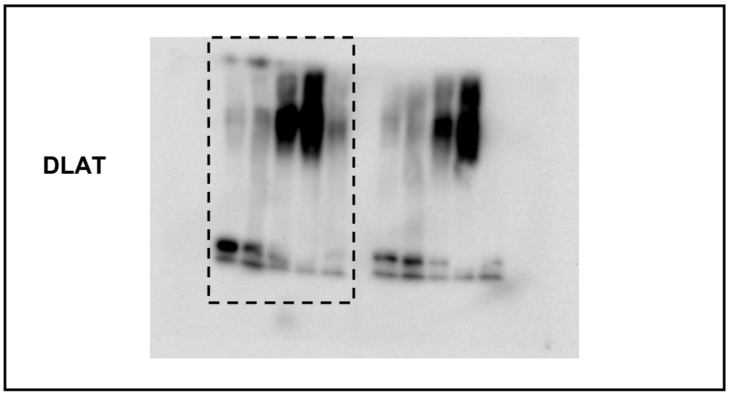

Supplement: Supplementary file 3 — Supporting File 3: advs76350‐sup‐0003‐DataFile.pdf. [file ADVS-9999-e76350-s001.pdf]
